# Iron Deficiency and Iron Deficiency Anemia: A Comprehensive Overview of Established and Emerging Concepts

**DOI:** 10.3390/ph18081104

**Published:** 2025-07-25

**Authors:** Bela Kolarš, Vesna Mijatović Jovin, Nemanja Živanović, Ivana Minaković, Nemanja Gvozdenović, Isidora Dickov Kokeza, Marija Lesjak

**Affiliations:** 1 Department of General Medicine and Geriatrics, Faculty of Medicine, University of Novi Sad, 21000 Novi Sad, Serbia; bela.kolars@mf.uns.ac.rs (B.K.); ivana.minakovic@mf.uns.ac.rs (I.M.); 2 Health Center “Novi Sad”, 21000 Novi Sad, Serbia; 3 Department of Pharmacology, Toxicology and Clinical Pharmacology, Faculty of Medicine, University of Novi Sad, 21000 Novi Sad, Serbia; 4 Department of Chemistry, Biochemistry and Environmental Protection, Faculty of Sciences, University of Novi Sad, 21000 Novi Sad, Serbia; nemanja.zivanovic@dh.uns.ac.rs (N.Ž.); marija.lesjak@dh.uns.ac.rs (M.L.); 5 Department of Emergeny Medicine, Faculty of Medicine, University of Novi Sad, 21000 Novi Sad, Serbia; 6 Clinic for Orthopedic Surgery and Traumatology, Clinical Center of Vojvodina, 21000 Novi Sad, Serbia; 7 Department of Gynaecology and Obstetrics, Faculty of Medicine, University of Novi Sad, 21000 Novi Sad, Serbia; 8 Clinic for Gynecology and Obstetrics, Clinical Center of Vojvodina, 21000 Novi Sad, Serbia

**Keywords:** iron deficiency, iron deficiency anemia, iron absorption, iron supplementation

## Abstract

Iron deficiency (ID) is the most prevalent micronutrient deficiency globally, affecting approximately one in four individuals, with a particularly high burden among children, women of reproductive age, and populations in low- and middle-income countries. It contributes significantly to the global burden of disease, with consequences ranging from impaired cognitive and motor development in children to increased risks during pregnancy, including low birth weight, preterm delivery, and maternal mortality, as well as reduced physical performance and quality of life in adults. ID often precedes iron deficiency anemia (IDA), though clinical and functional impairments—such as cognitive deficits, immune dysfunction, and fatigue—can occur even in the absence of anemia. Despite its widespread nature, challenges remain in precisely defining, diagnosing, and treating ID effectively. Advances in diagnostic tools allow for earlier detection, while novel therapeutic strategies, including updated oral dosing regimens and modern intravenous iron formulations, offer improved efficacy and tolerability. These approaches are particularly valuable in minimizing gastrointestinal side effects and enhancing patient adherence. This review is based on a comprehensive literature search conducted primarily through PubMed and Scopus, emphasizing studies published within the past 10–15 years. It is thematically structured to explore the epidemiology, health consequences, diagnostic complexities, and therapeutic developments related to ID. It highlights the multifactorial nature of ID and underscores the urgent need for early identification, targeted interventions, and updated clinical guidelines to reduce the long-term health and societal impacts of this preventable and treatable condition.

## 1. Introduction

Iron deficiency (ID) and iron deficiency anemia (IDA) are global health issues frequently encountered in daily clinical practice. ID is the most common nutritional deficiency worldwide, affecting up to 25% of the global population, or nearly 2 billion people [1]. The prevalence of ID is higher in developing countries compared to developed ones, although it is not limited to economically disadvantaged regions. In developing countries, ID typically arises from inadequate dietary intake of iron and is often associated with parasitic infections that cause bleeding. Additionally, malaria, HIV/AIDS, and tuberculosis contribute to high prevalence rates in some regions [2]. Children and women of reproductive age are particularly affected by ID due to increased iron requirements. ID arises when the body’s iron stores, particularly those in macrophages and hepatocytes, are depleted. Because most iron—approximately 25 mg daily—is used for hemoglobin (Hb) synthesis to support the production of around 200 billion red blood cells each day, IDA is the most evident consequence of ID. This often leads to the mistaken belief that ID and IDA are the same. However, ID is a broader condition that can precede the onset of IDA or impact other tissues beyond those involved in red blood cell production.

ID can be classified into three stages. In the first stage, which is characterized as mild ID, iron stores are depleted but the production of iron-dependent proteins is maintained. It was previously believed that the absence of iron stores had no adverse health effects. However, accumulating evidence suggests that even mild ID can lead to symptoms such as fatigue, cognitive impairment, reduced aerobic performance, compromised immune function, and poor sleep quality. The prevailing body of research strongly supports the need to prevent and manage even mild ID, not only in growing individuals and menstruating and pregnant women, but also in endurance athletes and the elderly to promote optimal health and development [3]. In the second stage, also known as iron-deficient erythropoiesis, the requirements for iron in erythropoiesis are no longer fully met, but Hb synthesis and erythropoiesis are maintained. Also, the production of iron-dependent proteins may be disrupted. The third stage is IDA, characterized by impaired Hb production. In IDA, erythrocytes are typically microcytic (smaller than normal red blood cells, with a mean corpuscular volume (MCV) below 80 fL) and hypochromic (paler due to reduced Hb content). However, in early stages, red blood cells may appear normocytic (normal size, MCV 80–100 fL) and normochromic before becoming microcytic as the deficiency worsens [1].

It is clear from the above that there is a hierarchy in how the body uses iron; erythropoiesis is protected when there is insufficient iron for all biological functions. Various systems in the body are affected by ID before erythropoiesis is compromised and anemia occurs. Besides its critical role in oxygen transport and storage, iron is essential for many important processes, including cellular respiration and energy production, the electron transport chain in mitochondria, DNA, RNA and protein synthesis, gene expression regulation, and cell proliferation and differentiation, among others [4].

## 2. Prevalence, Causes, and Outcomes of Anemia, ID, and IDA

Anemia is a condition characterized by a reduced number of red blood cells or a decreased concentration of Hb, resulting in diminished oxygen transport to body tissues. According to the World Health Organization (WHO), anemia affects 30% of non-pregnant women, 37% of pregnant women, and 40% of children under five worldwide [5]. Despite other causes like malaria, thalassemia, and sickle cell trait, ID remains the leading cause of anemia, making IDA the most common form of the condition. It is estimated that more than 1.2 billion people suffer from IDA worldwide, with prevalence varying significantly between low- and high-income countries [6,7].

In the least developed countries, particularly in sub-Saharan Africa, the prevalence of anemia among children aged 6–59 months often exceeds 60%, with some countries reporting rates as high as 86%. Data show that IDA affects up to 72.8% of children in certain regions (e.g., Ethiopian Somali region), and ID alone may affect over 90% in highly vulnerable subpopulations [7,8]. While national averages for IDA typically range from 12% to 46%, local studies in high-risk groups (e.g., breastfed infants or those exposed to infections and food insecurity) report extremely high rates, sometimes exceeding 60–70% [8].

In developing countries, anemia affects 20–39.9% of women of reproductive age in many regions, with some countries—such as Papua New Guinea and parts of Indonesia—reporting severe anemia prevalence exceeding 70%. Specific regional data point to anemia prevalence rates as high as 72.9% among women of reproductive age in Indonesia and up to 89.7% in Papua New Guinea, with a significant proportion attributable to ID and compounded by infections such as malaria and helminthiasis [9].

Although ID and IDA are often perceived as problems confined to low- and middle-income regions, recent evidence highlights their continued relevance and growing burden in high-income countries. Population-based studies in Europe have reported a consistent increase in IDA incidence from the early 2000s to the early 2010s, with some countries showing rates exceeding 12 per 1000 person-years. Women are particularly affected, with incidence rates up to four times higher than those in men. Prevalence data reflect a similar pattern, with overall rates approaching 3% and significantly higher values in females [10]. In the United States, data from the National Health and Nutrition Examination Survey (NHANES) between 1999 and 2018 indicate that 7.5% of pregnant women are anemic, while over 20% have ID based on serum ferritin levels. Among young children, anemia affects 4.7%, and ID reaches up to 30.5% when using physiologically relevant cutoffs [11]. These findings emphasize that ID and IDA remain underrecognized public health concerns in developed countries, disproportionately affecting women of reproductive age, young children, and certain ethnic minorities. Contributing factors may include demographic aging, increasing rates of obesity and chronic disease, medication use, and underdiagnosed gastrointestinal (GI) or gynecological conditions [11].

As previously mentioned, ID and IDA remain significant public health concerns across Europe, with notable age- and region-specific disparities. Earlier data indicated that 10–30% of women of reproductive age had inadequate iron stores, and 1.5–14% were affected by IDA. This was especially true in Northern European countries, where meat consumption tends to be lower [12]. However, newer data indicate that Eastern Europe now has the highest prevalence of ID and IDA across the continent, although exact figures are not consistently available [13]. A recent review of data from over 15 European countries revealed that 40–55% of women of reproductive age had small or depleted iron stores, defined by serum ferritin (SF) levels ≤ 30 μg/L. The prevalence of ID ranged from 10 to 32%, while IDA ranged from 2 to 5%, depending on the criteria used. Furthermore, 20–35% of these women had sufficient iron stores (SF > 70 μg/L) to complete a pregnancy without needing supplements. During pregnancy, particularly in the middle to late third trimester, the prevalence of ID and IDA increased significantly among women not receiving iron supplements, with ID affecting 28–85% and IDA affecting 21–35%. Women receiving iron supplementation had better iron status and a lower prevalence of ID and IDA [14]. The WHO Global Health Observatory data shows that anemia rates in pregnancy have remained largely unchanged over time, with rates decreasing from 41% in 2000 to only 37% in 2019 [15].

In children, a review of 44 studies conducted across 19 European countries revealed that 2–25% of infants aged 6–12 months were iron deficient, with higher rates among those from lower socioeconomic backgrounds and those who drank cow’s milk during their first year. For children aged 12–36 months, ID prevalence ranged from 3% to 48%, while the rate of IDA was as high as 50% in Eastern Europe, but less than 5% in Western Europe [16].

In Serbia, specific data on ID and IDA are limited. However, according to a UNICEF (United Nations International Children’s Emergency Fund) study conducted in 2000, nearly one in three children aged 6 to 60 months in Serbia suffered from anemia [17]. More recent data from the WHO indicates that in 2019, 19.5% of children aged 6–59 months in Serbia were anemic [15]. A 2013 study found that 23.7% of non-pregnant women aged 20–49 in Vojvodina, a northern region of Serbia, were anemic, compared to a national average of 27.7%. This research highlights anemia as a significant public health concern among non-pregnant women in Serbia, underscoring the need for continuous monitoring and control programs to reduce its high prevalence [18]. In Vojvodina, IDA ranks among the top ten conditions treated in day hospitals [19].

The WHO classifies the public health significance of anemia into four categories based on prevalence estimates in specific populations: “normal” (<5%), “mild” (5–19.9%), “moderate” (20.0–39.9%), and “severe” (≥40%) [20]. Given the magnitude of the issue, the WHO has set a target to reduce the prevalence of anemia among women of reproductive age, including adolescent girls, by 50% by the year 2030 [5].

The main causes of ID and IDA include low dietary iron intake and insufficient iron absorption during periods of life when iron requirements are particularly high, such as during periods of growth in children and adolescents, as well as during the reproductive years in women, especially during pregnancy and postpartum. Chronic GI bleeding, heavy menstrual bleeding, and malabsorption conditions like celiac disease and inflammatory bowel disease (IBD), where the integrity of the cells lining the GI tract is compromised, can also lead to IDA [21]. IDA occurs in 60–80% of patients with IBD, making it the most common extraintestinal complication of the disease, likely due to a combination of inflammation, impaired iron absorption, chronic blood loss, and dietary restrictions [22]. *Helicobacter pylori* is a spiral-shaped, Gram-negative bacterium that colonizes the gastric mucosa and is a common cause of chronic gastritis and peptic ulcers. Its infection is associated with ID and contributes to anemia through multiple mechanisms, including direct consumption of iron, GI blood loss from infection-induced lesions, and sequestration of iron-bound lactoferrin, reducing iron availability. Furthermore, chronic infection alters gastric physiology by decreasing acid secretion, impairing dietary iron absorption, while bacterial turnover further increases iron loss via stool excretion [23]. These factors together contribute to IDA, particularly in chronic infections. The use of nonsteroidal anti-inflammatory drugs can also cause stomach bleeding and affect iron status. Patients with chronic kidney disease (CKD) are also at increased risk due to impaired iron absorption, blood loss during dialysis, and elevated hepcidin levels, which restrict iron mobilization from stores [21]. Treatment with erythropoiesis-stimulating agents further elevates iron demand and can lead to functional ID if not monitored properly [1]. Similarly, obesity—especially following bariatric surgery—can impair iron status due to decreased gastric surface area, altered digestion, and low-grade inflammation that increases hepcidin production and limits iron absorption [24]. Postoperative anemia following major surgery is another important setting where ID may be unmasked or exacerbated, particularly when preexisting iron depletion has not been identified before surgery. Rare inherited forms of ID, such as iron-refractory IDA (IRIDA), caused by mutations in the transmembrane serine protease 6 (TMPRSS6) gene, which encodes the liver-expressed protease matriptase-2, should also be considered in cases resistant to oral iron therapy. These mutations lead to inappropriately high levels of hepcidin, the hormone that blocks intestinal iron absorption and iron release from stores, rendering oral iron therapy ineffective, though partial response to intravenous (IV) iron is possible. IRIDA is characterized by anemia from early childhood and represents a distinct genetic form of ID [21]. Polymorphisms in genes regulating iron transport and metabolism—such as solute carrier family 11 member 2 (SLC11A2), solute carrier family 40 member (SLC40A1), and transferrin (Tf)—may influence individual susceptibility to ID [25]. Furthermore, genetic disorders like thalassemia traits or congenital sideroblastic anemias can mimic IDA, underscoring the importance of differential diagnosis [26]. Recognition of these genetic contributors is essential, particularly in cases of unexplained or treatment-resistant anemia. Additional causes include chronic hematuria or intravascular hemolysis (e.g., in paroxysmal nocturnal hemoglobinuria), which lead to urinary iron loss, and bleeding disorders such as von Willebrand disease or hereditary hemorrhagic telangiectasia that contribute to recurrent, often unrecognized, blood loss [1].

Certain populations are at elevated risk for ID. Women, particularly adolescent girls on low-energy diets, are at high risk of ID. A study from Jordan found that the prevalence of ID and IDA was significantly higher among female university students who had attempted weight-loss diets compared to those who had not [27]. Vegetarians, particularly vegans, may also be at risk due to restrictive diets, along with voluntary blood donors and professional athletes [28]. For instance, 25% of British schoolchildren following a vegetarian diet had anemia, compared to 7% of children in the control group on a standard diet [12]. Other studies conducted on young women and adults have shown that vegetarians have lower iron stores compared to individuals who consume a mixed diet [29]. However, other studies show that well-planned vegetarian diets can support adequate iron stores [30]. Such diets include a variety of iron-rich plant-based foods such as legumes, tofu, nuts, seeds, whole grains, and iron-fortified products, paired with sources of vitamin C (e.g., citrus fruits, peppers, or tomatoes) to enhance iron absorption [22]. It is important to avoid consuming tea, coffee, or calcium-rich foods alongside iron-rich meals, as these can inhibit absorption. Timing iron intake with absorption enhancers is especially crucial in populations with higher requirements [8]. While both sexes can maintain adequate iron status on vegetarian diets if properly planned, premenopausal women—due to menstrual losses—require greater attention to iron density and absorption. Studies suggest that vegetarian women who follow these principles can maintain comparable iron status to omnivores [22].

Dietary habits vary significantly depending on an individual’s health status, age, and sex. Patients with chronic diseases such as autoimmune disorders, cardiovascular diseases, and diabetes mellitus often follow specific dietary recommendations tailored to manage their condition, which may inadvertently affect iron intake and absorption. Furthermore, physiological differences between age groups and sexes can modulate iron requirements and metabolism. Understanding how these diverse dietary patterns influence iron status is essential for designing effective nutritional interventions, particularly in populations at risk for ID and IDA. Individuals with autoimmune conditions such as celiac disease, IBD, and rheumatoid arthritis often follow restrictive diets due to GI intolerance or to manage inflammation. For instance, patients with celiac disease must adhere to a strict gluten-free diet, which can be low in iron-rich grains and cereals and often lacks iron fortification [31]. IBD patients may limit intake of fibrous foods during flare-ups, reducing their consumption of plant-based iron sources [32]. Additionally, chronic inflammation in these disorders impairs iron absorption by increasing hepcidin levels. Dietary recommendations for cardiovascular disease often emphasize reduced intake of red meat, saturated fat, and processed foods. While such diets are beneficial for heart health, they can reduce intake of heme iron, the most bioavailable form of dietary iron [33]. Furthermore, increased consumption of whole grains and legumes—though nutritionally valuable—can elevate phytate intake, which inhibits non-heme iron absorption [34]. These factors can potentially lower iron stores, particularly in individuals already at risk of deficiency. In people with type 2 diabetes, diets often emphasize whole grains, legumes, vegetables, and low glycemic index foods. These diets are rich in non-heme iron but also contain absorption inhibitors such as fiber and polyphenols. While iron overload is linked to increased diabetes risk, ID is associated with obesity—another major diabetes risk factor [35].

Additional risk factors, such as the type of contraception used by women, voluntary blood donation, or minor pathological blood losses (e.g., hemorrhoids, gynecological bleeding), can significantly hinder the ability to meet iron requirements [12].

Furthermore, it is estimated that nearly 15 million elderly individuals in the European Union may suffer from anemia, and the European Hematology Association has identified anemia in the elderly (AE) as a key research topic in a consensus document [36]. The current literature from large epidemiological surveys indicates that the etiology of AE can be broadly divided into three main categories: (1) nutritional deficiencies, mainly ID, but occasionally folic acid and vitamin B12 deficiencies, accounting for approximately 40–60% of cases; (2) anemia of inflammation (AI), which includes CKD, inflammatory or infectious diseases, and tumors; and (3) “unexplained” cases [37]. AE has been linked to a wide range of adverse outcomes, including frailty and reduced physical performance, diminished muscle strength and higher fall risk, cognitive decline and dementia, increased hospitalization rates and longer stays, and even a higher risk of mortality in longitudinal studies [38]. Importantly, these consequences are not limited to women. Although anemia is often underrecognized in men, recent data underscore its public health relevance, particularly among older males. A nationally representative survey in Malaysia found that 12.6% of men had anemia, with prevalence tripling in those over 60 years (30.7%) compared to younger men (10%) [39]. Older men are similarly affected by ID and anemia, particularly in the context of chronic diseases such as heart failure, cancer, and CKD, where low Hb levels and depleted iron stores are independently associated with worse clinical outcomes [40]. Studies have also shown that ID, even in the absence of anemia, can contribute to fatigue, impaired cognitive function, and decreased exercise tolerance in elderly males and females alike [38]. Moreover, frailty syndrome—which encompasses weakness, unintentional weight loss, and reduced endurance—has been strongly associated with both ID and IDA, suggesting that iron status plays a central role in maintaining functional independence in older adults [7]. Therefore, recognition and appropriate management of ID in both elderly men and women is crucial for mitigating physical decline, preserving cognitive abilities, and reducing morbidity [38]. This highlights the need for targeted screening protocols and individualized treatment strategies in this growing and vulnerable population.

ID and IDA are serious problems that negatively impact growth, cognitive and motor development, and behavior in children. They also increase susceptibility to infections, potentially raising the risk of mortality from severe infections during childhood, such as malaria [41]. Damage to psychomotor and mental development can be irreversible and may occur even in ID without IDA. Beyond adverse health outcomes, the condition may impair optimal developmental potential [42]. For instance, one study found that children who were iron deficient at birth showed reduced activation in brain regions associated with cognitive control at ages eight to eleven—even after their iron levels had been corrected [43]. In pregnant women, low iron levels can affect birth weight and may lead to preterm birth and even increase mortality rates for both mother and child.

Health problems can also arise in adults, including restless leg syndrome, fatigue and weakness, reduced intellectual and cognitive functions, difficulty concentrating, and decreased productivity at work, as well as depression. IDA reduces the work capacity of individuals and entire populations, with serious consequences for the economy and national development. Also, iron is necessary for the normal functioning of the brain, thyroid gland, immune system, and the production and metabolism of catecholamines and other neurotransmitters, as well as drug metabolism [21]. In older adults (>65 years), anemia is a recognized risk factor for hospitalization, unfavorable surgical outcomes, and higher all-cause mortality [44].

As a result, in 2021, anemia caused 52 million years lived with disability (YLDs) worldwide, accounting for 5.7% of all YLDs that year. Only low back pain and depressive disorders caused more disability. ID was the leading cause of anemia-related YLDs [7].

Some experts question whether ID is always problematic, especially when it occurs without symptoms or anemia. For example, one study found that women in clinical trials who were iron deficient and reported fatigue, experienced an improvement in energy levels after taking iron supplements. In contrast, the same intervention had no effect on the energy levels of women who were iron deficient but did not report fatigue [45]. This study suggests that, at least in adults, clinical illness related to ID can be improved with treatment. Nevertheless, in the absence of symptoms and with only low iron levels, it remains uncertain whether treatment would lead to any noticeable improvement. Overall, ID without anemia has been less extensively studied than IDA.

## 3. Iron Absorption and Metabolism

Iron homeostasis in the human body is tightly regulated; however, this regulation does not occur through excretion, but rather through the control of intestinal iron absorption, which plays a critical role in maintaining systemic iron balance. Namely, the human body lacks an active physiological mechanism for iron excretion; thus, daily iron losses through sloughing of intestinal epithelial cells, skin desquamation, menstruation, or minor bleeding are passive and not subject to homeostatic control. As a result, the regulation of systemic iron levels relies predominantly on the modulation of dietary iron absorption by enterocytes located in the proximal regions of the small intestine, particularly the duodenum and upper jejunum. These cells sense systemic iron needs and adjust iron uptake accordingly, making the intestinal epithelium the critical site for maintaining iron balance and preventing both deficiency and overload.

**Figure 1:**
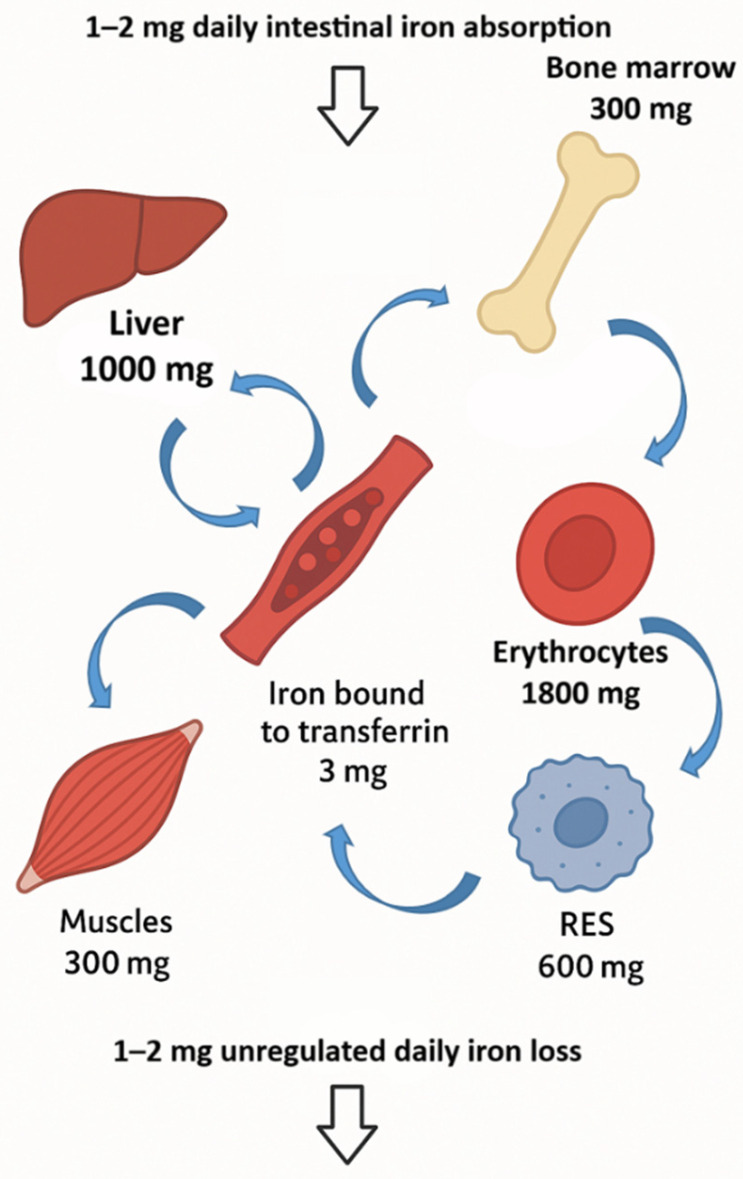
Distribution of iron in the human body. An adult human has approximately 4 g of iron in the body. Most of this iron, over 2 g, is found in hemoglobin, within erythroid precursors (300 mg) and mature circulating erythrocytes (1800 mg). The remaining iron is distributed as follows: 600 mg is present in the transit pool within reticuloendothelial macrophages, 1000 mg is stored in hepatocytes, and 300 mg is in muscles, within myoglobin. A smaller portion is present in plasma, bound to transferrin (3 mg), or integrated into various proteins and enzymes [46]. The daily dietary iron intake is approximately 10–20 mg, but only 1–2 mg is absorbed. The same amount is daily lost [47]. RES: reticuloendothelial system.

The total amount of iron in the human body is approximately 4 g, with over 60% of that iron found in erythrocytes and their precursors. The remaining iron is distributed across various compartments; approximately 600 mg resides in the transient pool within reticuloendothelial macrophages, while hepatocytes store around 1000 mg as a reserve that can be mobilized when the body’s demand for iron increases, as indicated by bidirectional arrows in Figure 1. Additionally, about 300 mg is found in muscle tissue as part of myoglobin, and a much smaller fraction circulates in the plasma, bound to transferrin (3 mg), or is incorporated into various iron-dependent proteins and enzymes [46]. Daily iron requirements are primarily met through endogenous sources, particularly the breakdown of circulating erythrocytes, with the majority being used for erythropoiesis (20–25 mg per day). Only about 5% of daily iron needs are absorbed from food, which compensates for the inevitable passive losses of iron ranging from 1 to 2 mg, as represented by the wide arrows in Figure 1 [47].

Daily iron requirements from food vary among individuals depending on age, sex, and physiological state. For women in their reproductive years, the daily requirement is 2.38 mg of absorbed iron, while women in the second and third trimesters of pregnancy require as much as 6.3 mg. In contrast, the average adult male needs about 1.4 mg. The average daily diet contains approximately 10–20 mg of iron, of which only 5 to 15% is absorbed [46]. This absorption rate depends on the amount of bioavailable iron in the food, as well as the individual’s condition: iron reserves, erythropoietic activity, and any presence of inflammation in the body. Under extreme conditions of ID, a theoretical maximum of 25 mg of elemental iron can be absorbed daily [48].

Interestingly, previous guidelines recommended iron supplementation in form of ferrous sulfate tablets of 200 mg, taken two to three times a day, to replenish iron in cases of IDA. Each of these tablets contains 65 mg of elemental iron, meaning that patients could be consuming up to 195 mg of elemental iron daily, which is far more than can be absorbed. Unabsorbed dietary iron passes through the small intestine and reaches the colon, where it is excreted in feces. Although unabsorbed iron does not enter systemic circulation, it is not biologically inert. In the colon, free iron can catalyze the formation of reactive oxygen species (ROS), contributing to oxidative stress and intestinal inflammation [49]. Moreover, excess unabsorbed iron alters gut microbiota composition by promoting the growth of pathogenic bacteria such as Escherichia coli while suppressing beneficial species like Lactobacillus and Bifidobacterium, leading to dysbiosis and increased risk of infections [50]. Furthermore, while iron absorption is generally well-regulated in healthy adults, studies suggest that this feedback control may be less effective in the elderly, increasing their risk of iron accumulation. Chronic excessive intake—especially from supplements or fortification—can lead to iron accumulation and, in genetically susceptible individuals, systemic iron overload with organ damage [51].

Nutrient bioavailability refers to the fraction of nutrients from food that is absorbed and effectively utilized by the body. Iron absorption varies significantly depending on the food source, with reported rates of 25–30% from organ meats, 7–9% from leafy greens, 4% from grains, and just 2% from dried legumes [52]. These differences suggest that the type of food consumed, along with other dietary factors, plays a crucial role in determining iron bioavailability.

Iron in the diet exists in two forms: non-heme and heme iron. Non-heme iron, found in both plant and animal foods, constitutes as much as 90% of the total iron consumed through diet. However, it has low biological availability, and its absorption is significantly influenced by the presence of promoters and inhibitors of iron absorption in food. Heme iron, on the other hand, is found only in animal-based foods. Although it makes up only about 10% of the iron consumed by individuals who eat meat, its higher bioavailability can contribute to more than 40% of the total absorbed iron. Its absorption is minimally affected by inhibitors in food [47].

**Figure 2:**
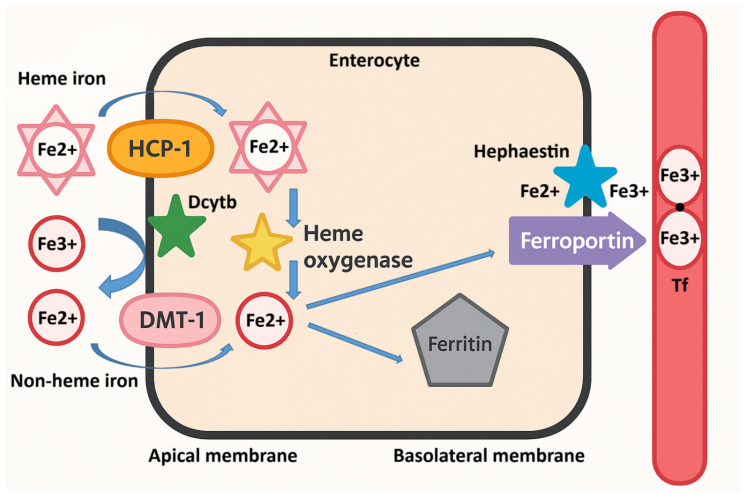
Iron absorption. Non-heme iron must be in the Fe^2+^ state before entering the enterocyte. Duodenal cytochrome b (Dcytb) reduces Fe^3+^ to Fe^2+^, after which divalent metal transporter 1 (DMT-1) transports Fe^2+^ into the enterocyte [53]. Heme iron is transported into the enterocyte cytoplasm via heme carrier protein 1 (HCP-1) transporter, where heme oxygenase removes the heme iron from the protoporphyrin ring. In the cytoplasm, iron can bind to ferritin for storage or be transported out of the enterocyte via ferroportin. Hephaestin then oxidizes the iron into its ferric (Fe^3+^) form, allowing it to bind to transferrin (Tf) in the blood for transport throughout the body [54].

The absorption of non-heme iron occurs as follows; after the reduction in ferric iron (Fe^3+^) to ferrous iron (Fe^2+^) by duodenal cytochrome b (Dcytb) on the apical membrane of enterocytes, iron is transported into the enterocyte via divalent metal transporter 1 (DMT-1), as illustrated in Figure 2. Reducing agents in the diet, such as ascorbic acid, lactic acid, citric acid, and other organic acids, as well as foods that stimulate the production of endogenous gastric acid, also aid in the reduction of Fe^3+^ to Fe^2+^ and enhance iron absorption. DMT-1 is not specific to iron; therefore, other divalent metal ions competitively inhibit its absorption [53].

When there is a need for iron in the body, it is exported from enterocytes into circulation across the basolateral membrane via the ferroportin (FPN) transporter and is oxidized to the Fe^3+^ form by the transmembrane feroxidase hephaestin (Heph), as represented by the blue star in Figure 2. It then binds to the serum glycoprotein Tf for transport to peripheral tissues. Each apotransferrin molecule (Tf without iron) can bind two Fe^3+^ atoms, which are transported by circulation to cells that have Tf receptors, such as immature erythroid cells in the bone marrow. Tf has a high affinity for iron and under physiological conditions in healthy individuals, its saturation is 25–30%, resulting in a negligible amount of free iron unbound to Tf in the plasma. Fragments of Tf receptors on cells are released into circulation and their serum concentration is proportional to the body’s iron needs [54].

Excessive iron intake, particularly when it exceeds the body’s storage and transport capacity, can lead to the presence of non-transferrin-bound iron (NTBI) in the circulation [55]. Unlike iron that is safely bound to Tf or ferritin, NTBI is redox-active and can catalyze the formation of ROS. These cause oxidative damage to cellular components, including lipids, proteins, and DNA, contributing to tissue injury and the pathogenesis of several chronic conditions such as cardiovascular disease, liver fibrosis, diabetes, and neurodegeneration [55]. The risk is particularly relevant in individuals with genetic predispositions to iron overload (e.g., hereditary hemochromatosis) or in the elderly. Therefore, iron supplementation should be carefully monitored and tailored to individual needs to avoid potential toxicity.

Erythropoiesis is stimulated by the hormone erythropoietin, which is produced in the kidneys in response to hypoxia or anemia. Approximately 7 days after the initial stimulation of erythroid precursors in the bone marrow, erythropoiesis culminates in the release of reticulocytes [54]. These immature red blood cells mature in the circulation for about 24 h, during which they acquire the morphological characteristics of erythrocytes, becoming smaller and losing remnants of their nuclei. Reticulocytes typically constitute about 1% of the mass of red blood cells. In the formation of Hb, heme (iron bound to protoporphyrin) combines with globin. In a state of ID, zinc replaces iron in the final stages of erythropoiesis, so the level of zinc protoporphyrin (ZPP) in erythrocytes can also be used diagnostically as a marker of ID or IDA [54].

When there is no need for iron in the body, it is retained in enterocytes in the form of ferritin—a soluble protein complex that can store up to 4500 iron atoms per molecule—and is removed into the intestinal lumen along with the enterocyte [56]. In this way, duodenal enterocytes act as a short-term iron reservoir for a duration of 3 to 5 days, moderating excess iron absorption from the time of their migration and differentiation from stem cells at the base of the intestinal crypt until they are shed from the top of the villus into the intestinal lumen [57].

The key regulator of iron homeostasis in the body is hepcidin—a peptide hormone produced primarily in the liver and, under certain conditions, in other tissues. Its synthesis increases with elevated iron levels in the plasma and decreases in cases of anemia and hypoxia. Since there is no active mechanism for eliminating iron from the body, hepcidin plays a crucial role in protecting against iron overload. When iron is not bound to proteins, its ability to change oxidation states makes it toxic and capable of generating free radicals. Maintaining iron levels within narrow limits is vital, as both ID and iron overload can lead to severe hematological, metabolic and neurodegenerative disorders, as well as carcinogenesis. Hepcidin acts by inhibiting the release of iron from cells by binding to FPN, the only known iron exporter, inducing its degradation. This way, it inhibits iron absorption from the intestines into the bloodstream, the recycling of iron from macrophages and the mobilization of iron from liver stores. Hepcidin is also produced in increased amounts during infection and inflammation, mediated by cytokines (primarily IL-6); this leads to a reduction in plasma iron levels, making it unavailable to microorganisms and contributing to the development of AI [58]. This type of anemia is resistant to oral iron therapy, because the elevated levels of hepcidin cause iron to be trapped in enterocytes. It is believed that the mechanism of limiting iron availability to invasive microorganisms serves as a defense strategy for the body, known as “nutritional immunity” [59]. By restricting access to iron, which is essential for their active metabolism, the growth and proliferation of microorganisms are inhibited [60]. In addition to iron, the body also limits the availability of other essential metals like zinc as part of this same protective process [59]. Many bacteria depend on these metals to grow and survive, so by sequestering them, the body effectively slows down infection. Several key host proteins involved in metal withholding have already been identified, but ongoing research continues to uncover additional players [61]. At the same time, bacteria have evolved sophisticated strategies to counter these defenses, allowing them to locate and acquire trace metals even under restrictive conditions. For example, certain bacterial proteins such as zinc-regulated iron-regulated gene A (ZigA) may act as metallochaperones, helping shuttle limited metal supplies to enzymes that are crucial for bacterial survival and host colonization [60]. Gaining a deeper understanding of how bacteria detect and respond to metal scarcity—through changes in gene and protein expression—may open up new possibilities for targeted therapies, particularly in individuals with conditions like IDA, where metal balance is already disrupted.

Given the critical importance of maintaining iron balance and the absence of a physiological excretion mechanism, the body relies on tightly regulated systemic and cellular mechanisms to fine-tune iron availability. These regulatory layers ensure adequate iron supply for erythropoiesis and metabolism, while protecting tissues from iron-induced toxicity.

As mentioned, hepcidin acts by binding to FPN, which is predominantly expressed on key iron-transporting cells such as duodenal enterocytes, splenic and hepatic macrophages, and hepatocytes. Upon binding to FPN, hepcidin triggers its internalization and degradation and also directly blocks the export channel, thereby reducing iron efflux into plasma [61]. This mechanism allows the body to restrict dietary iron absorption and limit the recycling of iron from senescent red blood cells under conditions of iron sufficiency or overload. In contrast, ID leads to a strong suppression of hepcidin synthesis, facilitating increased iron absorption in the intestine and mobilization of iron from tissue stores. The suppression of hepcidin during ID involves multiple overlapping pathways. A key regulator is the bone morphogenetic protein (BMP)–SMAD signaling pathway (named after the Sma and Mad genes in worms and flies), which normally activates hepcidin transcription in hepatocytes [62]. In iron-deficient states, this pathway is downregulated due to reduced production of BMP6 by liver sinusoidal endothelial cells, cleavage of the BMP coreceptor hemojuvelin by the protease TMPRSS6 (matriptase-2), and loss of transferrin receptor 2 from the hepatocyte surface due to reduced diferric Tf levels [63]. Collectively, these changes blunt the BMP–SMAD signal and thereby suppress hepcidin expression.

In addition to transcriptional regulation, epigenetic mechanisms contribute to hepcidin suppression. For instance, histone deacetylase 3 removes transcription-activating histone marks from the hepcidin promoter region, reinforcing low hormone production in iron-depleted states [64].

Another important regulatory axis involves erythroferrone (ERFE), a hormone secreted by erythroblasts in response to erythropoietin stimulation, particularly during anemia and hypoxia [65]. ERFE acts as a suppressor of hepcidin to ensure that iron supply meets the demands of increased erythropoiesis.

At the local level, iron absorption is enhanced through activation of hypoxia-inducible factor 2 alpha (HIF-2α) in enterocytes. HIF-2α upregulates the expression of DMT-1 and Dcytb—both involved in apical iron uptake from the intestinal lumen—as well as FPN, which mediates basolateral export into circulation [66].

Macrophages, particularly those in the spleen and liver, play a critical role in recycling iron from aging red blood cells through the activity of heme oxygenase-1. However, in ID, the Hb content of erythrocytes is reduced (hypochromia), leading to a diminished total iron yield from recycling. Interestingly, increased expression of FPN on erythrocyte membranes during deficiency may serve as a compensatory mechanism to maintain circulating iron levels [67].

These systemic mechanisms ensure that iron absorption and mobilization are adjusted according to physiological demand. Yet iron balance is also regulated at the cellular level, where post-transcriptional and autophagic processes control intracellular iron distribution, storage, and utilization.

While systemic iron levels are primarily regulated by the hormone hepcidin, cellular iron regulation is governed by an intricate post-transcriptional network centered around iron-regulatory proteins (IRPs). These proteins—IRP1 and IRP2—modulate the expression of key iron metabolism genes by binding to conserved RNA stem-loop structures known as iron-responsive elements (IREs), located in the untranslated regions (UTRs) of target mRNAs [68]. In iron-deficient conditions, IRPs bind to IREs in the 3′ UTRs of transcripts such as transferrin receptor 1 and DMT-1, stabilizing the mRNAs and promoting iron uptake. Conversely, binding to IREs in the 5′ UTRs of transcripts encoding ferritin, FPN, aminolevulinate synthase 2, and HIF2α inhibits their translation, conserving iron by reducing storage and limiting iron-dependent pathways [65].

To prevent iron overload in certain cells, alternative regulatory mechanisms operate independently of IRPs. For example, enterocytes and erythroblasts express a FPN isoform lacking a 5′UTR IRE, allowing iron export to continue even under conditions where IRPs are active [67]. In parallel, mechanistic target of rapamycin (mTOR) pathway inhibition during iron scarcity triggers the RNA-binding protein tristetraprolin, which downregulates transferrin receptor 1 and FPN expression to conserve intracellular iron for essential metabolic functions [69].

Cells can also reclaim stored iron via a selective autophagic process called ferritinophagy, in which ferritin is targeted to autophagosomes by nuclear receptor coactivator 4. This process is upregulated in ID and supports iron supply for erythropoiesis, as demonstrated in vitro and in animal models [70]. Despite the clinical use of SF as a biomarker of iron status, the physiological mechanisms underlying its secretion and potential roles in circulation remain incompletely understood.

Iron restriction limits early erythroid progenitor expansion and enhances iron efficiency during terminal erythropoiesis. ID reduces erythropoietin responsiveness by inactivating the iron-dependent enzyme aconitase, lowering isocitrate production—an effect reversible with iron or isocitrate [71]. In ID without anemia, erythropoietin levels remain normal, but terminal erythropoiesis is altered; apoptosis decreases and late erythroblasts accumulate [72].

With anemia or hypoxia, erythropoietin levels rise sharply, stimulating mediators like ERFE, growth differentiation factor 15, and platelet-derived growth factor-BB to suppress hepcidin and increase iron availability [73]. As erythroblast numbers rise and iron remains limited, heme synthesis declines and globin translation is inhibited via heme-regulated inhibitor kinase (HRI) activation and downstream mTOR suppression [73]. This coordination produces microcytic, hypochromic erythrocytes and helps conserve iron, though it may not prevent iron depletion in other tissues.

Metabolomic shifts in ID include elevated levels of lactate, pyruvate, and tricarboxylic acid (TCA) cycle intermediates, such as succinate, reflecting a shift toward anaerobic glycolysis and impaired mitochondrial oxidative metabolism [65,66,67]. Additionally, iron-regulated amino acids (e.g., histidine and tryptophan), which serve as cofactors for iron-dependent enzymes, show altered plasma or intracellular concentrations [66,67]. A decline in ATP levels and increased production of ROS may also occur, indicating mitochondrial dysfunction and redox imbalance [55].

ID triggers a coordinated network of molecular adaptations at the genomic, proteomic, and metabolomic levels, involving key regulators of systemic and cellular iron homeostasis, as described above and summarized in Table 1. These adaptations include transcriptional and post-transcriptional regulation of iron-related genes via IRPs, altered expression of iron transporters (e.g., DMT-1, FPN), storage proteins (e.g., ferritin), and hormonal regulators (e.g., hepcidin), as well as changes in metabolites involved in oxidative stress, energy metabolism, and erythropoiesis [65,66,67]. Together, these changes represent a highly regulated adaptive response aimed at maximizing iron absorption, mobilization, and utilization to maintain iron availability for essential physiological functions—particularly erythropoiesis—despite reduced iron supply.

**Table 1:** Genes, proteins, and metabolites altered in iron deficiency (ID).

Name	Type	Function/Mechanism	Change in ID	Functional Consequence	Reference
Hepcidin	Gene/Protein	Regulates systemic iron homeostasis by degrading ferroportin	Downregulated	Increases iron absorption and release from stores	[61]
Hemojuvelin	Gene/Protein	BMP6 co-receptor, activates BMP–SMAD pathway for hepcidin induction	Downregulated	Reduces hepcidin expression, increases iron absorption	[63]
BMP6	Gene/Protein	Stimulates hepcidin via SMAD signaling	Downregulated	Reduces hepcidin synthesis	[63]
Transferrin Receptor 2	Gene/Protein	Iron sensor in hepatocytes; mediates hepcidin regulation	Downregulated	Decreases hepcidin synthesis, enhances iron absorption	[63]
Erythroferrone	Protein/Hormone	Suppresses hepcidin in response to erythropoietin	Upregulated	Increases iron availability for erythropoiesis	[65]
Histone Deacetylase 3	Epigenetic Regulator	Epigenetically represses hepcidin expression	Increased activity	Decreases hepcidin transcription, enhances absorption	[64]
mTOR	Protein/Signaling	Regulates cell growth, autophagy, erythropoiesis; Fe-sensitive	Inhibited (decreased)	Promotes ferritinophagy and metabolic adaptation	[69]
Tristetraprolin	Protein/RNA-binding	Binds to mRNAs, promotes degradation; regulates inflammatory and iron metabolism mRNAs	Upregulated	Modulates inflammatory signals affecting hepcidin and iron regulation	[69]
Ferroportin	Gene/Protein	Iron exporter in enterocytes/macrophages	Stabilized (increased)	Enhances plasma iron availability	[67]
DMT-1 (SLC11A2)	Gene/Protein	Imports dietary non-heme iron into enterocytes	Upregulated	Promotes intestinal iron absorption	[65,66,67]
Dcytb	Gene/Protein	Reduces Fe^3+^ to Fe^2+^ at brush border	Upregulated	Facilitates iron absorption via DMT-1	[65,66,67]
Transferrin receptor 1	Gene/Protein	Cellular uptake of transferrin-bound iron	Upregulated	Increases cellular iron import	[65]
Transferrin	Protein	Iron transport in plasma	Decreased or unchanged	Affects iron delivery to tissues	[63]
SLC11A1	Gene/Protein	Iron transporter in macrophages	Upregulated	Modulates immune-related iron handling	[25]
Ferritin (FTH1/FTL)	Gene/Protein	Cytosolic iron storage	Downregulated	Reflects depleted iron stores	[70]
NCOA4	Gene/Protein	Mediates ferritin degradation (ferritinophagy)	Upregulated	Mobilizes stored intracellular iron	[70]
IRP1/IRP2	Protein	Iron sensors; regulate translation via IREs	Active (increased)	Enhances iron uptake, suppresses storage/export	[68]
HRI	Protein/Kinase	Senses heme deficiency, phosphorylates eIF2α to inhibit protein synthesis	Activated	Prevents accumulation of unassembled globin; modulates erythropoiesis under ID	[73]
5-aminolevulinic acid synthase 2	Gene/Protein (Enzyme)	Catalyzes first step in heme biosynthesis in erythroid cells; essential for hemoglobin production	Downregulated or functionally impaired due to iron shortage	Limits heme and hemoglobin synthesis, contributing to anemia and ineffective erythropoiesis	[65]
Erythropoietin	Gene/Protein	Stimulates red blood cell production	Upregulated	Compensates for anemia	[73]
HIF-2α	Gene/Protein	Controls hypoxia response; induces DMT-1/Erythropoietin	Upregulated	Enhances erythropoiesis and absorption	[66]
Ferritin (circulating)	Protein/Metabolite	Stored iron in blood	Decreased	Key diagnostic marker of ID	[70]
Lactate	Metabolite	End product of anaerobic glycolysis	Increased	Suggests metabolic shift due to hypoxia	[65,66,67]
Succinate	Metabolite	TCA cycle intermediate	Accumulates	Activates HIFs and IRPs	[65,66,67]
Pyruvate	Metabolite	Glycolysis end product	Increased	Reflects altered cellular metabolism	[65,66,67]
ATP	Metabolite	Energy currency of the cell	Decreased	Reduced mitochondrial function	[65,66,67]
Reactive oxygen species	Metabolite	By-product of redox metabolism	Variable (increased)	Indicates oxidative stress	[55]
Iron-regulated amino acids (e.g., histidine, tryptophan)	Metabolites	Cofactors for Fe-dependent enzymes	Altered	Affected by Fe-dependent metabolic pathways	[65,66,67]

This table summarizes key molecular alterations observed during ID across genomic, proteomic, and metabolomic levels. It includes genes, proteins, and metabolites that are either upregulated or downregulated in response to low iron availability. Each entry lists the molecule, its function or mechanism of action, and the functional consequence of its altered expression or activity. Together, these changes represent an integrated adaptive response aimed at maintaining iron homeostasis, supporting erythropoiesis, and mitigating the metabolic consequences of iron scarcity. BMP6: bone morphogenetic protein 6; BMP–SMAD: bone morphogenetic protein–SMAD signaling pathway regulating hepcidin expression; mTOR: mechanistic target of rapamycin; DMT-1: divalent metal transporter 1; Dcytb: duodenal cytochrome b; SLC11A1: solute carrier family 11 member 1; NCOA4: nuclear receptor coactivator 4; IRP1/IRP2: iron-regulatory proteins 1 and 2; IRE: iron-responsive element; HRI: heme-regulated inhibitor kinase; HIF-2α: hypoxia-inducible factor 2 alpha; TCA cycle: citric acid cycle or Krebs cycle; FTH1 (ferritin heavy chain 1) and FTL (ferritin light chain) are the two subunits of ferritin, the primary intracellular iron storage protein.

Iron absorption is a tightly regulated physiological process influenced by both the chemical form of iron in the diet and the presence of various dietary components. While heme iron from animal sources is generally well absorbed, non-heme iron from plant-based foods requires conversion and favorable conditions in the GI tract to be efficiently taken up [74]. The efficiency of iron absorption is not only dependent on the intrinsic properties of iron itself but is also significantly modulated by other compounds present in the diet. These compounds can either enhance or inhibit iron absorption by altering its solubility, redox state, or interaction with intestinal transporters [74,75,76]. Understanding the role of these dietary promoters and inhibitors is essential for developing nutritional strategies to optimize iron bioavailability, particularly in populations at risk for ID.

### 3.1. Inhibitors of Iron Absorption in Food

Phytates, which act as phosphorus storage molecules in plants, form complexes with iron and are regarded as the primary inhibitors of non-heme iron absorption from food. The negative effect of phytates on iron absorption is dose-dependent and begins at very low concentrations of 2–10 mg per meal. Certain food preparation methods, such as milling, heat treatment, soaking, germination, fermentation, addition of ascorbic acid or enzyme phytase, and even genetic engineering can remove or break down phytates, thereby partially or completely eliminating their negative impact on non-heme iron absorption. People who consume a monotonous diet of unrefined grains like wheat and rice—often the poorest individuals in the world—frequently suffer from mineral deficiencies. To improve iron absorption from simple meals made with grains or legumes (that do not have ingredients that help with iron absorption), the molar ratio of phytate to iron should be <1:1, or preferably <0.4:1. In mixed meals that include vegetables with vitamin C or meat (which help absorb iron), a higher phytate level is acceptable—up to six times the amount of iron [74].

The impact of phytate on iron absorption, as observed in single-meal studies (which investigate the effects of a single food intake on iron absorption in a controlled setting), is often seen as overstated when compared to its influence within a whole diet [75]. For example, Cook et al. compared iron absorption from single meals with absorption from a regular diet and they found that absorption from single meals is not a good reflection of how the body absorbs iron from a typical diet [76]. Similarly, Hoppe et al. found no significant difference in iron status between individuals consuming low-phytate whole grain bread and those consuming high-phytate whole grain bread as part of a whole diet [77]. According to the authors, a likely explanation for the findings is that participants adjusted their diets, altering the balance between iron absorption inhibitors and enhancers. Additionally, in a mixed diet, increasing wholegrain intake in women already consuming a high-wholegrain diet does not seem to impair iron status.

Conversely, another study reported that phytate consumption reduced the bioavailability of iron and calcium in the diets of pregnant women [34]. However, other research suggests that a diet consistently high in phytates may gradually diminish their inhibitory effect on non-heme iron absorption, particularly in young women with low iron levels. The authors suggest further research to explore potential mechanisms [78].

Polyphenols are secondary plant metabolites that encompass a wide range of structurally diverse compounds, with flavonoids being one of the largest groups of plant phenols. They play a crucial role in plant interactions with the environment, reproduction, and defense. They are regularly consumed in significant amounts through diet of plant origin, such as vegetables, fruits, and certain grains and legumes, as well as tea, coffee, and wine. Flavonoids are important not only for their effect on iron absorption, but also for their various other impacts on the human body. Beyond protecting biomolecules from free radicals, flavonoids are involved in many biochemical processes in the body. As a result, they offer a range of health benefits, including lowering blood pressure, reducing low-density lipoprotein (LDL) cholesterol levels, decreasing the risk of cardiovascular diseases, inhibiting carcinogenesis and neurodegeneration, and reducing platelet aggregation and inflammation [79].

The exact mechanism by which polyphenols inhibit the absorption of non-heme iron is not yet fully understood, but it is strongly believed that their ability to chelate iron is responsible for this effect [80,81]. Their effect on iron absorption depends on both the quantity and the type of polyphenols consumed. Furthermore, several studies have shown that flavonoids have a significant impact on hepcidin levels in vivo [58,74]. Flavonoids modulate hepcidin through diverse and sometimes opposing mechanisms. Baicalein, for example, increases hepcidin levels in mice with anemia by boosting certain proteins in the liver [82]. Genistein, a compound found in soy, also strongly increases hepcidin in the body, possibly by turning on certain genes involved in iron regulation [83,84]. On the other hand, myricetin works differently by reducing hepcidin and increasing the levels of FPN, which raises iron levels in the blood [85]. One study showed that administration of quercetin, a polyphenolic compound found in fruits and vegetables, to mice increased hepcidin expression, which correlated with changes in serum iron levels and transferrin saturation [86]. Another study found that prenatal exposure to quercetin led to increased hepatic iron storage and upregulation of inflammatory cytokines in adult mice, possibly via epigenetic changes, while also reducing oxidative DNA damage despite no apparent effects on fetal erythropoiesis [87]. Lesjak et al. found that both oral and intraperitoneal administration of quercetin reduced serum and tissue iron levels in rats by impairing iron absorption and downregulating key duodenal iron transporters, while also inducing hepatic hepcidin expression [35]. These findings highlight quercetin’s potential to influence systemic iron metabolism and suggest a possible role in managing iron-related disorders, as summarized in Table 2.

**Table 2:** Summarizes selected flavonoids, their primary dietary sources, their modulatory effects on iron metabolism, proposed mechanisms of action, and potential relevance to iron-related disorders.

Flavonoid	Source (Fruits/Vegetables/Grains)	Modulation of Iron	Mode of Action	Disease Relevance	References
Quercetin	Onions, apples, berries, grapes	Inhibits absorption	Chelates iron; inhibits FPN; affects IRP/IRE system	Avoid in IDA; potential for iron redistribution therapy	[81]
Epicatechin	Tea, cocoa, apples	Promotes bioavailability (in vitro)	May increase Fe^2+^ availability	Potentially beneficial in iron deficiency	[88]
Kaempferol	Kale, beans, tea, broccoli	Promotes bioavailability (in vitro)	May increase Fe^2+^ availability	Potentially beneficial in iron deficiency	[88]
Myricetin	Berries, grapes, tea	Inhibits hepcidin expression	Inhibits BMP/SMAD pathway	May counteract hepcidin overexpression	[85]
Genistein	Soybeans	Increases hepcidin	Activates hepcidin expression (unknown pathway)	Useful in iron overload disorders	[83]
Ipriflavone	Synthetic (from daidzein)	Increases hepcidin	Synthetic analog, promotes hepcidin	Possible role in iron metabolism disorders	[84]
Icariin	*Epimedium* (Chinese herbs)	Increases hepcidin	Activates Stat3 and Smad1/5/8 pathways	Regulates iron homeostasis; potential therapeutic use	[89]

FPN: ferroportin; IRP: iron-regulatory protein; IRE: iron-responsive element; BMP/SMAD pathway is the main regulator of hepcidin expression.

Research has shown that ascorbic acid can neutralize the inhibitory impact of low polyphenol concentrations, but this effect diminishes when polyphenol levels are high [90].

Calcium negatively affects the absorption of both non-heme and heme iron, which distinguishes it from other inhibitors that only impact the absorption of non-heme iron. Although the exact mechanism by which calcium exerts its inhibitory effect remains unclear, several potential explanations have been suggested. It has been proposed that calcium might hinder the final phase of iron transport from mucosal cells to plasma, following the convergence of heme and non-heme iron within a shared intracellular pool—potentially due to their distinct apical mucosal receptors [91]. Conversely, an alternative perspective suggests that despite differences in apical receptors, calcium’s inhibitory effect could take place at the very onset of iron absorption by interfering with its entry into mucosal cells, even at concentrations commonly found in dietary intake [92]. However, research indicates that prolonged calcium supplementation, whether consumed with or apart from meals, has no significant impact on iron levels. The discrepancy between the short- and long-term effects of calcium on iron absorption is likely due to the body’s adaptive mechanisms. With sustained calcium intake, the body adjusts iron absorption processes to maintain iron homeostasis [93]. This could explain how infants and the elderly—whose diets are frequently rich in milk and dairy products, which are high in calcium—may experience impaired iron absorption. Infants rely almost exclusively on milk—either breast milk or formula—while older adults often consume dairy as a practical and calcium-rich food source. Infants maintain adequate iron status during the first six months through the highly bioavailable iron in breast milk and iron stores accumulated prenatally [94]. The early introduction of cow’s milk, however, which is low in iron but rich in calcium, casein, and whey proteins, can both inhibit iron absorption and contribute to microscopic GI bleeding, thus increasing the risk of ID. Complementary feeding with iron-rich or fortified foods after six months is essential to meet rising iron demands [46]. Elderly individuals, meanwhile, may consume dairy frequently for practical reasons such as ease of digestion and bone health support, but often face additional factors impairing iron status, including hypochlorhydria (reduced stomach acid production) and chronic conditions causing increased iron loss or reduced absorption [39]. Given that calcium inhibits the absorption of both non-heme and heme iron, concerns arise about how these vulnerable populations maintain adequate iron status without compromising calcium intake. If calcium hinders iron transport, particularly at the final phase of mucosal cell-to-plasma export, the question becomes how iron requirements are still met. One likely answer lies in the body’s adaptive response; with sustained calcium intake, iron absorption pathways adjust to preserve iron homeostasis [93]. This may involve altered expression or regulation of iron transporter proteins or changes in the sensitivity of enterocytes to dietary components. In addition, bioavailability enhancers such as ascorbic acid can help counteract calcium’s inhibitory effects, and while evidence on long-term benefits is mixed, separating calcium and iron intake may help reduce competition in some individuals [39]. Ultimately, calcium modulates—but does not prevent—iron absorption, and with appropriate dietary planning, it is possible to ensure sufficient intake of both nutrients even in milk-dependent populations [95]. Further studies are needed to clarify calcium’s exact mechanism of action and to develop targeted strategies that reduce nutrient–nutrient interference.

Certain proteins have been shown to inhibit iron absorption, including those found in milk, soybeans, and eggs, as well as albumin, casein, and whey [96].

### 3.2. Promoters of Iron Absorption in Food

Ascorbic acid is one of the primary dietary enhancers of iron absorption. It enhances non-heme iron absorption primarily by reducing Fe^3+^ to Fe^2+^, facilitating its transport via DMT-1. It forms a chelate with ferric iron in the acidic environment of the stomach, keeping it soluble in the alkaline pH of the duodenum, the first section of the small intestine. In the duodenum, ascorbic acid donates an electron to ferric ions, reducing them to their absorbable ferrous form while also functioning as a free radical scavenger. This facilitates iron uptake by allowing ferrous ions to cross the brush border membrane of enterocytes [97]. Numerous in vivo and in vitro studies have investigated the effect of ascorbic acid on iron absorption. A recent study concluded that adding ascorbic acid to the digestate of plant products enhanced iron absorption in the Caco-2 cell culture model [98]. A separate study utilized an in vitro digestion/Caco-2 cell model to examine how phytic acid, sodium oxalate, and sodium silicate influence non-heme iron bioavailability in the presence or absence of ascorbic acid. The results demonstrated that these compounds hinder ferrous iron absorption; however, ascorbic acid mitigates their inhibitory effects and promotes iron uptake [99]. A different study also found that ascorbic acid can counteract the inhibitory effects of polyphenols, supporting similar results [100]. Ascorbic acid’s effect on iron absorption is dose-dependent and occurs only when both nutrients, ascorbic acid and iron, are consumed together [101]. The amount of ascorbic acid required to have a positive effect on non-heme iron absorption is approximately 30–100 mg per day, which aligns with the recommended daily intake [102]. It has been reported that while 500 mg of ascorbic acid taken with food increases iron absorption six-fold, taking ascorbic acid 4–8 h before a meal is less effective [103]. In this regard, adding ascorbic acid to the diet—especially one rich in iron—may appear effective for increasing iron intake. However, aside from the technical challenges in preparation and storage due to ascorbic acid’s instability, its addition to the whole diet has a more negligible impact on enhancing iron absorption. This is because its effectiveness depends largely on timing and direct interaction with the meal, rather than simply being present in the diet. While ascorbic acid has a positive effect on non-heme iron absorption after a single meal, chronic supplementation with ascorbic acid has not been shown to improve iron status in humans. The underlying reason for this discrepancy is not yet fully understood [104].

Animal tissues like beef, chicken, fish, pork, and lamb enhance non-heme iron absorption. Studies have shown that adding meat, liver, or fish to plant-based meals can significantly increase iron uptake, likely by inactivating luminal inhibitors and facilitating iron transport [105]. Even small amounts of meat, such as 50 g of pork or 25 g of beef, have been found to improve non-heme iron bioavailability [106]. Fish consumption has also been shown to enhance iron absorption from phytate-rich meals [107]. Additionally, organ meats like kidney, heart, and lung, when fortified into infant cereals, have demonstrated a substantial increase in non-heme iron absorption [108]. The exact mechanism behind this “meat factor” remains unclear, but proteins, cysteine-containing peptides, glycosaminoglycans, and L-α-glycerophosphocholine may contribute to its effect by reducing and chelating iron [109,110].

Prebiotics are nondigestible food components, primarily certain fibers and oligosaccharides, that are selectively utilized by beneficial gut microorganisms, thereby conferring health benefits to the host [111]. Common dietary sources of prebiotics include inulin, fructo-oligosaccharides, galacto-oligosaccharides, garlic, onions, leeks, asparagus, and bananas [110]. Probiotics, on the other hand, are live microorganisms—such as Lactobacillus and Bifidobacterium species—that, when administered in adequate amounts, confer health benefits by modulating the gut microbiota [112]. Typical probiotic foods include yogurt, kefir, sauerkraut, kimchi, and other fermented products [112,113,114,115,116,117].

Prebiotics have been extensively investigated for their potential to enhance iron absorption. Although this possibility had been considered in the past, only recent human studies using stable iron isotopes have provided solid evidence supporting a positive effect. The magnitude of this effect varies and is influenced by numerous factors, including the type and dosage of the prebiotic, whether intake is short- or long-term, the form of iron administered, and the age and iron status of the individuals studied [112,113,114,115]. These variables complicate the ability to make broadly applicable conclusions. Nonetheless, among the compounds studied so far, galacto-oligosaccharides and fructo-oligosaccharides appear most effective, particularly when paired with ferrous fumarate at doses above 3.5 g [116].

The beneficial effect of prebiotics on iron absorption is thought to involve multiple biological pathways. These may include slowing gastric emptying, which provides more time for iron to become soluble; activating specific genes in intestinal cells that facilitate iron uptake; encouraging the growth of enterocytes, thereby expanding the surface available for nutrient absorption; and fostering the microbial production of short-chain fatty acids, which acidify the distal intestine and enhance iron solubility [116]. Additionally, prebiotics may improve iron availability through chelation or reduction, while also exerting anti-inflammatory effects within the gut and potentially throughout the body [117].

Probiotics may also support iron status by improving gut barrier function, reducing intestinal inflammation, and modulating the microbiota composition in ways that favor iron absorption [116]. Together, prebiotics and probiotics contribute to a healthier gut environment, which can be advantageous in managing ID.

## 4. Diagnostic Indicators of ID and Anemia

From a diagnostic standpoint, anemia is defined as a Hb level below the lower limit of normal values for a given population and according to the laboratory conducting the testing. According to the WHO, this value is defined as Hb below 120 g/L for non-pregnant women and below 130 g/L for men [118]. Determining whether anemia is caused by ID is an important step in guiding appropriate diagnostic evaluation. In the Western world, excluding the increased iron needs of infants and adolescents, ID is primarily caused by bleeding or pregnancy [22]. In adult males or postmenopausal females, unexplained ID requires investigation, with colon cancer assumed until proven otherwise.

Since the signs and symptoms are nonspecific and often absent, the initial suspicion of ID typically arises from laboratory findings, such as microcytic or normocytic anemia, prompting further diagnostic evaluation. However, diagnosing ID through laboratory tests is challenging due to the dynamic nature of iron homeostasis. No single test can reliably assess iron absorption, transport, storage, and utilization. Therefore, a comprehensive medical history and clinical examination are important for accurately interpreting laboratory results.

**Figure 3:**
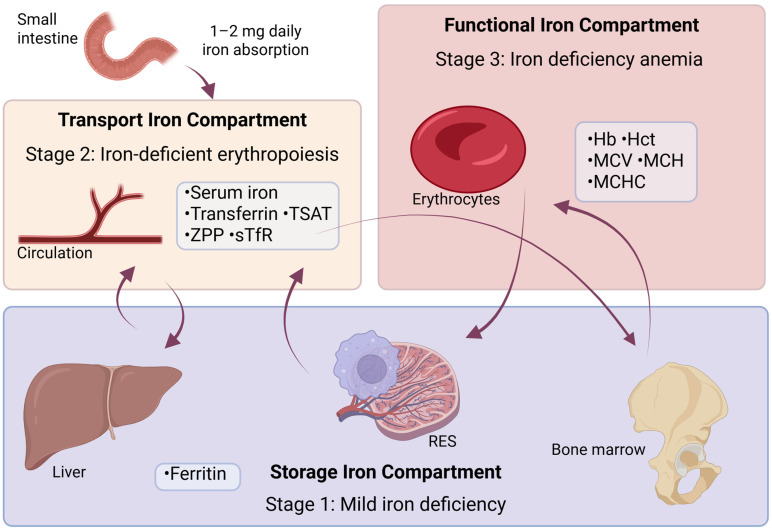
Body iron compartments and the parameters for their assessment. Following absorption, the body’s iron is divided into three main compartments: storage, transport, and functional. As ID advances, these compartments are progressively affected, with specific diagnostic markers used at each stage. Storage Iron: Iron is mainly stored as ferritin in liver cells and macrophages, with serum ferritin reflecting tissue levels. Deficiency becomes evident when stores are depleted, but red blood cell production remains unaffected [119]. Transport Iron: This compartment is primarily assessed using TSAT. Around half of the body’s iron is allocated for red blood cell production. Once transport iron is depleted, iron-deficient erythropoiesis occurs, though Hb levels remain normal [120]. Functional Iron: As deficiency progresses, available functional iron decreases, leading to IDA. Hb is the main marker. TSAT: transferrin saturation; ZPP: zinc protoporphyrin; sTfR: serum soluble transferrin receptors; Tf: transferrin; TfR: transferrin receptor; Hb: hemoglobin; Hct: hematocrit; MCV: mean corpuscular volume; MCH: mean corpuscular Hb; MCHC: mean corpuscular Hb concentration; RES: reticuloendothelial system. Created with BioRender.com.

There are three main compartments of iron in the body that describe ID, as shown in Figure 3: storage iron, transport iron, and functional iron. Functional iron refers to the portion of total body iron actively involved in essential processes, primarily oxygen transport and cellular metabolism. This iron is found in Hb, myoglobin, and enzymes involved in cellular respiration and energy production.

Depletion of each compartment leads to different stages of ID as represented in Table 3.

Short-term variations in physiological iron needs are met by the release of iron reserves, primarily stored as intracellular ferritin, mostly in hepatocytes and specialized macrophages, as shown in Figure 3. Ferritin is the primary protein responsible for storing iron in tissues, with a small portion present in serum, making it available for measurement. SF represents a small portion of the total ferritin pool in the body, but the concentration of SF reflects the amount of iron reserves, as shown in Table 3. When the iron reserves are depleted, the first stage of ID is reached, but there are still no consequences for erythropoiesis [119].

The iron supplied by the transport compartment is primarily intended for red blood cell production, as the iron requirement for erythropoiesis is much greater than that of other tissues. If the supply can no longer be met, the second stage of ID is reached—iron-deficient erythropoiesis, without a significant decrease in Hb concentration; however, the production of iron-dependent proteins may be reduced [120]. Indicators that provide information about the adequacy of iron supply include transferrin saturation (TSAT), zinc protoporphyrin (ZPP), and serum soluble transferrin receptor (sTfR) concentrations. These are illustrated in Figure 2.

The third stage of ID, IDA, is marked by a decrease in the functional iron compartment—most notably reflected in a reduction in Hb concentration, as shown in Figure 3. The various assays used to assess iron and its stores will be reviewed.

SF is the most useful marker for ID. SF levels below 15 µg/L strongly suggest ID [121]. Levels up to 30 µg/L may still indicate deficiency, though with lower specificity [122]. Reference ranges can differ depending on the analytical method and patient population, so local standards should be established and regularly updated. A low SF level combined with a low TSAT is enough to confirm ID. If Hb levels are also low, IDA can be diagnosed. SF acts as an acute-phase protein, meaning its levels increase in response to inflammation, as well as in conditions like kidney disease, liver disease, and malignancy. Studies have found that SF levels should be interpreted with caution in obese individuals, as evidence suggests that SF may reflect inflammation rather than accurately indicate iron status in this population [123]. To more reliably assess ID, a complete iron profile—including transferrin—is preferred over SF alone [124]. While formulas for interpreting SF levels in the presence of elevated inflammatory markers have been suggested, the supporting evidence is not yet strong enough to apply a “corrected” assessment of iron status in clinical practice [125]. SF levels within the laboratory reference range do not rule out ID when inflammatory markers are elevated or there is a history of acute or chronic illness, so additional investigation may be needed.

Serum iron refers to the fraction of iron that circulates primarily bound to Tf, ready for incorporation into Hb within maturing erythroblasts in the bone marrow. Iron that is not bound to Tf accounts for less than 1% of the total iron in serum and is usually not detected in most routine analyses [126]. An individual with ID will usually exhibit a low concentration of serum iron, but it is a dynamic parameter. The serum iron level relies on the effective recycling of iron by tissue macrophages from aging erythrocytes, as well as on the iron absorbed from the diet [125]. Under normal circumstances, this process is highly efficient but can fluctuate significantly and rapidly during inflammation. With a transferrin-bound iron pool of around 3 mg, this pool must be replenished six to eight times daily to support normal erythropoiesis [127]. Therefore, while serum iron follows a natural daily variation, it is also influenced by external factors that can cause sudden changes. As a result, no single serum iron measurement can definitively diagnose ID and should only be used to calculate TSAT [128].

Total iron-binding capacity (TIBC) (expressed in μg/dL or μmol/dL) is the maximum amount of iron that Tf can bind when it is 100% saturated. TIBC is determined by adding excess iron to serum, removing any unbound iron, and then measuring the iron concentration in the remaining sample.

TSAT represents the percentage of occupied iron-binding sites on all Tf molecules and is calculated as the ratio of serum iron to Tf or as the ratio of serum iron to TIBC. TSAT depends on serum iron concentration, so it shares the same variability and lack of specificity, with diurnal variation—that is, fluctuations in iron levels throughout the day—which can reach up to 70% [129]. Conditions like malnutrition and chronic disease reduce Tf synthesis, increasing TSAT and limiting its utility. A definitive diagnostic threshold for TSAT is not well-established, but some guidelines suggest <20% as a screening threshold [130]. A lower target of <16%, which is supported by some of the literature, is considered more specific [131].

Tf, the main protein responsible for iron transport in plasma, can be quantified using immunological methods. Both TIBC and Tf levels increase in states of ID, as presented in Table 3. However, as a negative acute-phase reactant, Tf levels may decrease during inflammation. While TIBC and Tf measurements are more stable than serum iron levels, their specificity remains limited [132].

During the final stages of Hb production, iron is incorporated into porphyrin. When iron availability is reduced, zinc is used instead, leading to the formation of ZPP, which can be detected, as demonstrated in Table 3. The use of ZPP as a marker for ID is uncertain. High levels can also be found in conditions like functional ID, lead poisoning, and disorders that promote excess porphyrin production, such as thalassemia [133]. In simple ID, it offers no more insight than SF. Its lack of specificity is evident in multifactorial anemia, as seen in studies where malaria and hemoglobinopathies were present alongside ID [134].

The amount of Tf receptor on a cell is proportional to its iron needs. The proteolytic product of the Tf receptor circulates in the plasma as sTfR. The concentration of sTfR in circulation is directly related to the total concentration of cellular Tf receptor [132]. Since most iron is used by erythroid precursors, the level of sTfR in circulation correlates with the mass of erythroid precursors (i.e., the rate of erythropoiesis). The level of sTfR increases in ID, when cells need to be more competitive in acquiring iron, as shown in Table 3. sTfR has been proposed as an alternative to traditional ID tests. However, more recent evidence suggests it is not useful for detecting early-stage ID and is more relevant in advanced cases, offering no clear advantage over SF [135]. Elevated levels are also observed in conditions involving erythroid hyperplasia, such as hemolysis, megaloblastic anemia, thalassemia, and hypoxia [136]. Interestingly, during inflammation or iron-restricted erythropoiesis, sTfR levels do not increase. This makes TfR measurements valuable for differentiating between true ID and inflammatory conditions that result in low serum iron and low TSAT, such as AI [137].

The full blood count (FBC) confirms anemia if Hb is below the laboratory reference range; however, Hb alone does not indicate iron status. Automated FBC analysis includes mean corpuscular volume (MCV), mean corpuscular Hb (MCH), and mean corpuscular Hb concentration (MCHC), which may decrease in ID but lack consistent diagnostic value. A decrease in MCH and MCV, as well as an increase in the number of hypochromic erythrocytes with an MCH less than 28 pg, occurs relatively late due to the lifespan of erythrocytes (around 120 days) [21]. While ID typically causes microcytic, hypochromic anemia, low values can also occur in conditions like thalassemia. If ID coexists with vitamin B12 or folic acid deficiency, MCV may be high, normal, or low. MCV remains normal in up to 40% of iron-deficient patients and in mixed hematinic deficiencies [138]. Red cell distribution width (RDW) measures red blood cell size variation. An elevated RDW indicates anisocytosis, reflecting increased size variation on a blood smear. A high RDW can signal early iron, folate, or B12 deficiency, rising before other RBC parameters. It also helps differentiate IDA (high RDW, normal/low MCV) from heterozygous thalassemia (normal RDW, low MCV), though further testing is needed for confirmation [139]. The Hb content in reticulocytes can indicate changes in erythropoiesis. An early decline can occur after erythropoiesis-stimulating agent therapy, while an early increase can be seen after iron supplementation [21].

Hepcidin is produced in the liver in response to iron levels, inflammation, and erythropoietic demand [140]. Inflammation increases hepcidin, reducing iron release from macrophages, enterocytes, and hepatocytes, while ID suppresses its synthesis, as evidenced by Table 3 [141]. Hepcidin shows potential as a diagnostic tool for iron status and response to oral iron, though factors like diurnal variation can affect its accuracy [142]. Hepcidin concentrations are typically lowest in the early morning, gradually rise throughout the day (from approximately 7:30 to 19:30), and then decline during the night, reflecting a characteristic diurnal rhythm [143]. Hepcidin assays, primarily using mass spectrometry or radioimmunoassay, offer good specificity but limited sensitivity at low serum levels [144]. Their high cost and low throughput restrict routine clinical use, though they may help establish reference standards [145]. Efforts are ongoing to improve standardization and normal ranges. Recently, ELISA-based methods have emerged as simpler alternatives, though still costly. While few commercial assays exist, the development of hepcidin-25 (bioactive) ELISA holds promise for future clinical applications. Hepcidin may help distinguish ID from AI and predict responsiveness to oral iron therapy. However, its full integration into clinical practice and public health requires further efforts to standardize assays, evaluate the significance of specific hepcidin isoforms, establish clinical decision thresholds, and ensure the widespread availability of validated testing methods [146].

**Table 3:** Biochemical markers of iron status [132].

	Stage I	Stage II	Stage III	Compartment
Ferritin	↓	↓↓	↓↓	Storage
Iron	N	↓	↓	Transport
Transferrin	N	↑	↑	Transport
TIBC	N	↑	↑	Transport
TSAT	N	↓	↓	Transport
sTfR	N	↑	↑	Transport
ZPP	N	↑	↑	Transport
Hb	N	N	↓	Functional
MCV	N	N	↓	Functional
RDW	N	N	↑	Functional
Hepcidin	N	↓	↓	NA

The table summarizes key laboratory markers across the three stages of iron deficiency (ID) and iron deficiency anemia (IDA): Stage I (mild ID), Stage II (iron-deficient erythropoiesis), and Stage III (IDA). Arrows indicate relative changes compared to normal values: ↓: Low level; ↓↓: Severely low level; ↑: High level; N: Normal level. The markers are grouped by physiological compartments—storage (ferritin) and transport (serum iron, transferrin) (TIBC: total iron-binding capacity; TSAT: transferrin saturation; sTfR: soluble transferrin receptor; ZPP: zinc protoporphyrin)—and functional iron compartment (Hb: hemoglobin; MCV: mean corpuscular volume; RDW: red blood cell distribution width). Hepcidin levels, which regulate iron homeostasis, decrease during stages II and III. This profile aids in differentiating stages of ID progression.

In IDA without inflammation iron stores, transport iron and functional iron are all reduced. As soon as iron supplies for erythropoiesis become insufficient, Tf production increases to enhance iron transport, Tf receptor production is boosted to facilitate iron delivery to cells, leading to an increase in serum sTfR, and instead of Hb, ZPP is produced. There is a decrease in ferritin, serum iron and TSAT, while TIBC increases [132]. These changes are summarized in Table 3.

### Diagnostic Challenges in Anemia of Inflammation

In chronic inflammatory diseases, ID presents differently than in pure IDA due to the complex interplay between inflammation, iron metabolism, and erythropoiesis. This condition—referred to as AI or anemia of chronic disease—is characterized by functional ID. Although iron stores (e.g., SF) may be normal or elevated, iron is sequestered in macrophages and unavailable for erythropoiesis due to hepcidin-mediated inhibition of iron absorption and export. Consequently, AI typically manifests as a normocytic, normochromic anemia, whereas IDA presents as microcytic, hypochromic anemia. Additionally, in AI, serum ferritin is often elevated (due to its role as an acute-phase protein), while TSAT and serum iron are low [147]. This contrasts with IDA, where both ferritin and TSAT are low. Diagnosis is further complicated when AI coexists with absolute ID, as inflammatory markers mask the classic signs of IDA. Inflammatory cytokines not only restrict iron availability but also suppress erythropoietin production and impair erythroid progenitor response, further distinguishing AI from classical IDA. Moreover, the shortened red blood cell lifespan in AI contributes to anemia independently of iron status [148]. Overall, while both types share symptoms like fatigue and reduced exercise tolerance, AI often presents insidiously, is less severe, and is commonly underrecognized or misattributed to the underlying disease [147].

Treatment strategies for AI differ from those used in IDA due to their distinct underlying mechanisms. While IDA is primarily managed with oral iron supplementation, AI is characterized by iron sequestration driven by inflammation-induced hepcidin upregulation, which limits the efficacy of oral iron. Therefore, IV iron is often required in AI, particularly in cases of concurrent true ID [147]. Additionally, erythropoiesis-stimulating agents are frequently employed in AI, especially in patients with CKD or cancer. Crucially, effective management of AI depends on addressing the underlying inflammatory condition. Emerging therapies that target hepcidin or its upstream regulators offer promising new avenues for AI-specific treatment.

In mobile blood donation units and field-based health interventions, point-of-care Hb measurement devices play a critical role in rapidly screening individuals for anemia or eligibility for blood donation. Two common approaches are portable hemoglobinometers, and noninvasive spectrophotometry devices. Portable hemoglobinometers typically use a small drop of capillary or venous blood to determine Hb concentration via photometric analysis. These devices offer high accuracy, especially when venous samples are used, and show good agreement with laboratory-based reference methods [149]. Their main advantages are portability, speed, and reliability, making them suitable for field use. However, they are invasive, require trained personnel, and involve consumables (e.g., cuvettes, lancets), which may increase cost and complexity in large-scale screening. On the other hand, noninvasive spectrophotometry devices estimate Hb levels without drawing blood, using occlusion spectroscopy—a technique that temporarily halts blood flow in the finger and analyzes the light transmission spectrum [121]. These devices are painless, reduce biohazard risks, and are easier to operate. However, their accuracy is lower, particularly in certain populations (e.g., females or those with low Hb), and results are affected by factors like skin tone, temperature, and perfusion [149]. Studies consistently show that spectrophotometry devices tend to overestimate hemoglobin and have wider variability, potentially leading to inappropriate donor deferral or acceptance. Overall, while noninvasive methods offer logistical and donor-related advantages, portable invasive devices remain the preferred choice when accuracy is critical—especially near regulatory decision thresholds or in populations with high anemia prevalence.

Recent advances in artificial intelligence have introduced novel approaches for non-invasive anemia screening. One study explored the use of machine learning models—including convolutional neural networks (CNN), naïve Bayes, decision trees, k-nearest neighbors, and support vector machines—to detect IDA based on images of the conjunctiva, fingernails, and palmar skin [150]. All models were trained, validated, and tested using labeled image datasets, with performance compared across anatomical regions. Among the evaluated methods, the CNN consistently demonstrated superior accuracy in detecting anemia, particularly when using images of the palm. The palmar region proved to be the most practical and reliable anatomical site, especially in young children, due to its ease of access and higher image quality, as opposed to the conjunctiva, which may pose examination challenges and carry a risk of discomfort or infection during inspection. These results support the use of CNN-based tools in resource-limited settings where clinical personnel and diagnostic equipment are scarce. The study underscores the potential of deep learning approaches to enhance early anemia detection, especially in low-resource environments such as rural areas of Ghana. While promising, implementation remains limited by the lack of user-friendly interfaces. Future development aims to integrate these models into mobile applications to facilitate widespread clinical and community-based use.

Although there is no single universal guideline for diagnosing ID and IDA, there is broad international consensus on key diagnostic principles, as reflected in recommendations from several authoritative bodies. The WHO provides global public health guidance, particularly for population-level screening and interventions. It defines anemia using Hb thresholds and identifies SF as the primary marker of iron status, with adjustments for inflammation using markers such as C-reactive protein (CRP) and alpha-1-acid glycoprotein [147]. For example, SF levels below 15 µg/L in adults indicate depleted iron stores, while a threshold of <30 µg/L is used when inflammation is present [151].

The European Hematology Association (EHA) recommends a more comprehensive diagnostic approach: Hb, SF, TSAT, sTfR, and inflammatory markers [152]. Their guidelines address both individual diagnosis and broader population health strategies, while also offering interpretation strategies in the context of inflammation.

The Centers for Disease Control and Prevention (CDC) focuses primarily on public health monitoring and screening, especially among children and women of reproductive age. Like WHO, the CDC recommends assessing Hb and SF levels, with adjustments for inflammation [11].

The British Society for Haematology and other national bodies also provide clinical practice guidelines tailored to specific healthcare settings, yet their diagnostic frameworks are generally aligned with those of WHO and EHA.

In summary, while no single universal guideline exists, the diagnostic criteria for ID and IDA are largely standardized across leading health organizations. There is strong agreement on the use of low Hb levels to define anemia and low SF (adjusted for inflammation) to indicate ID. Complementary markers such as TSAT, sTfR, CRP, and the reticulocyte Hb content enhance diagnostic accuracy, particularly in complex or inflammatory conditions. Differences between guidelines are mostly limited to specific threshold values and contextual emphasis depending on whether the focus is clinical or public health, or whether the setting is in a developed or developing country.

If IDA is confirmed, it is important to determine the cause as soon as possible, as heterogeneous studies have found that 8–15% of patients over 60 years old have GI cancer as the underlying cause [153,154].

For confirmed IDA, the British Society of Gastroenterology recommends the following tests: urinalysis, screening for celiac disease, and, when appropriate, endoscopic examination of both the upper and lower GI tract [155]. Celiac disease is present in 3–5% of IDA cases and a routine screening is recommended, either through serological testing or a small intestine biopsy during a gastroscopy. Age, gender, Hb concentration, and MCV are independent risk predictors for GI cancer in cases of IDA and should be considered as part of a comprehensive risk assessment. At present, fecal immunochemical testing for occult blood is not recommended for risk assessment in patients with IDA [119]. However, since guidelines are subject to change, this recommendation may be modified in the future. Gastroscopy and colonoscopy are the first-line GI investigations for men and postmenopausal women with newly diagnosed IDA [154]. Both are diagnostic methods for direct visualization; gastroscopy examines the upper GI tract, while colonoscopy evaluates the colon and rectum using a flexible endoscopic tube. For those who are not suitable for colonoscopy, CT colonography is a reasonable alternative. Young women with IDA rarely have GI pathology as the cause of anemia, so after screening for celiac disease, further investigation is recommended only if there are additional clinical concerns [155].

For patients with IDA and no identifiable etiology after bidirectional endoscopy, non-invasive testing for *Helicobacter pylori* is recommended, followed by treatment if the result is positive [119].

## 5. Addressing Iron Deficiency: Replacement Therapy and Prevention

The three most common approaches to addressing ID and increasing iron absorption are changing eating habits—by improving the variety, nutritional value, and bioavailability of iron in the diet; supplementation—taking higher doses of iron independently of meals; and fortification—adding iron to food during processing or through biofortification. These methods can be used independently or in combination. Addressing IDA across different age groups requires a tailored yet harmonized approach that considers specific physiological demands, dietary patterns, and risk factors [48]. Infants and young children, due to rapid growth and limited iron reserves, benefit most from iron-rich complementary feeding, fortified foods, and micronutrient powders—single-dose sachets containing a blend of essential vitamins and minerals that are added to home-prepared foods to prevent and treat micronutrient deficiencies [5]. Adolescents and women of reproductive age require strategies that meet increased iron needs from menstruation, pregnancy, or growth—such as targeted supplementation and culturally appropriate dietary guidance [156]. Adults and older individuals often face challenges related to chronic diseases, inflammation, or impaired absorption, necessitating the use of well-tolerated iron formulations, food fortification, and regular monitoring [38]. Despite these population-specific differences, common solutions exist: improving dietary diversity with iron-rich and absorption-enhancing foods, reducing intake of iron inhibitors, using low-dose or alternate-day iron supplementation to improve adherence and minimize side effects, and implementing food fortification programs using widely consumed staples [157]. When adapted to regional dietary patterns and supported by public health education, these strategies can synergistically improve iron status across the life course and ensure both individual and population-level benefits [156].

### 5.1. Changes in Eating Habits

Dietary habits should be adjusted to increase the consumption of foods rich in both heme and non-heme iron, along with absorption enhancers, while reducing the intake of iron absorption inhibitors. It is well known that iron bioavailability is just as important as the total amount of iron in the diet. Even though it showed significant practical limitations, a change in dietary habits is the favored way of treating IDA. Apart from the difficulty of changing an individual’s dietary preferences, food rich in highly bioavailable iron, such as meat, is expensive, especially in developing countries.

Oysters, liver, lean red meat (especially beef), dark poultry, lamb, pork, and shellfish are excellent animal-based options. Fish like tuna and salmon also provide a good amount of iron [26].

Although some studies suggest that meat is the superior source of iron and that vegetarian diets may increase the risk of ID, other research reports that a well-balanced vegetarian diet can provide sufficient iron. Key sources include legumes (e.g., lima beans, soybeans, kidney beans, dried beans, and peas), fortified or whole grains (e.g., wheat, millet, oats, and brown rice), dried fruits (e.g., prunes, raisins, and apricots), nuts, seeds, and green vegetables (e.g., broccoli, spinach, kale, collards, asparagus, and dandelion greens) [30]. Moreover, research has shown that in Western countries, individuals following a healthy vegetarian diet do not have a higher incidence of IDA than omnivores [158].

Dairy products and eggs are poor sources of iron and can reduce its absorption. Caseins in milk, along with certain forms of calcium, have been shown to inhibit iron uptake. Additionally, infants with cow’s milk allergies may be particularly vulnerable to intestinal blood loss due to the irritating effects of dairy products [95]. Studies indicate an inverse relationship between iron status, measured as SF, and higher dairy consumption in toddlers, especially when dairy displaces iron-rich or iron-enhancing foods [159]. Eggs, particularly yolks, have also been found to hinder iron absorption [160].

Fruits and vegetables aid the absorption of non-heme iron. They contain vitamin C and organic acids that keep iron in a reduced form, increasing absorption of non-heme iron when consumed in the same meal [161].

Among plant-based foods, certain vegetables and legumes stand out as significant sources of dietary iron. Based on standard portion sizes, the highest iron concentrations among vegetables were found in cooked spinach (6.4 mg iron per cup), Jerusalem artichokes (5.1 mg per cup), and various legumes such as lima beans, hyacinth beans, and soybeans (ranging from 4.4 to 4.9 mg per serving) [162]. Other leafy greens including Swiss chard, amaranth leaves, beet greens, and chrysanthemum leaves provided between 2.7 and 4.0 mg of iron per cup, making them valuable contributors to daily iron intake.

The American Red Cross highlights strawberries, watermelon, raisins, dates, figs, prunes, and dried peaches or apricots as particularly good fruit sources of iron. Incorporating these iron-rich plant foods—especially when combined with vitamin C sources like citrus fruits or berries—can help optimize non-heme iron absorption and support iron status [163].

Tea, coffee, and cocoa should be avoided during meals if ID is suspected, as the polyphenols and tannins in these beverages interfere with the absorption of non-heme iron. Black tea, in particular, seems to have the strongest inhibitory effect [158].

Promoting dietary improvement and diversification, particularly by increasing the intake of bioavailable iron from animal products, fruits, and vegetables rich in vitamin C, is the only approach that can sustainably improve iron status. Supplementation and fortification alone are not enough. Encouraging the consumption of micronutrient-rich foods improves overall health, supports sustainable solutions, fosters long-term behavioral changes in high-risk groups, and is often linked to income-generating activities [164]. However, specific recommendations should be tailored to age groups, as well as regional and local variations in diet, taking into account the availability of foods, demographic characteristics, and cultural preferences [9]. These factors significantly influence both the types of iron-rich foods accessible to populations and the feasibility of meeting recommended iron intakes.

#### Region-Specific Strategies for Improving Iron Nutrition

A region-specific approach to iron nutrition ensures that dietary recommendations are not only scientifically sound but also culturally acceptable and practical for the target population.

The types of iron-rich foods available in each region vary based on local agricultural practices and dietary habits. For instance, in Argentina, where red meat consumption is high, heme iron intake is typically adequate, whereas in India, where vegetarianism is prevalent due to religious and cultural beliefs, diets are predominantly plant-based and rely on non-heme iron from legumes, lentils, and grains [165]. In Japan, seafood and seaweed provide significant iron sources, while in sub-Saharan African countries like Nigeria, dark leafy vegetables, millet, and beans are staple iron sources [166]. These regional dietary patterns necessitate customized guidelines that align with existing habits.

Additionally, cultural preferences play a key role in determining food choices, which should be considered to improve the acceptance and effectiveness of dietary recommendations. For example, in Sri Lanka, where tea is commonly consumed with meals, public health messaging may focus on separating tea consumption from iron-rich meals to avoid absorption inhibition [97]. Similarly, regions where lacto-vegetarianism is practiced—such as Nepal or parts of South India—may benefit from guidelines emphasizing plant-based iron sources and enhancers like vitamin C-rich fruits (e.g., guava, papaya) [111].

Iron levels in food can also be influenced by soil quality and farming practices. In parts of Southeast Asia, where soils may be depleted in iron, staple crops such as rice can have low iron content, making it challenging to meet recommended intake through food alone. In such cases, agricultural interventions such as biofortification—for example, iron-fortified rice in Bangladesh and the Philippines—can play an important role [167]. Guidelines in such regions can recommend the consumption of biofortified foods to address ID.

Furthermore, seasonal variations in food availability should be accounted for, with guidelines recommending iron-rich foods that are in season or locally available to ensure a consistent intake of iron throughout the year [167]. Effective dietary guidelines should be accompanied by public health campaigns that raise awareness about the importance of iron and educate populations about ways to enhance iron absorption.

The WHO has launched several initiatives to promote dietary improvements as a means of preventing and treating ID. One notable example is the Anaemia Action Alliance, established in collaboration with UNICEF, which aims to coordinate and accelerate global efforts to reduce anemia through strategies such as dietary modification, food fortification, and iron supplementation [168]. Complementing this effort, WHO has published practical tools and guidelines, including the manual Iron Deficiency Anaemia: Assessment, Prevention and Control and the comprehensive resource Nutritional Anaemias: Tools for Effective Prevention and Control (2017). These documents provide evidence-based strategies for addressing ID through improved dietary intake, fortification programs, and targeted supplementation, with specific guidance for different population groups [169]. The WHO’s educational materials also emphasize the importance of consuming iron-rich foods—such as meat, legumes, and fortified cereals—alongside vitamin C-rich foods to boost iron absorption [170]. Together, these resources support countries in developing effective, context-appropriate interventions to reduce the global burden of IDA.

Given the high bioavailability of heme iron, its excessive intake—especially from red meat—raises concerns about long-term health effects. Emerging evidence links elevated heme iron consumption to several chronic diseases, as summarized below [171].

Among the most consistently associated conditions is colorectal cancer, for which high heme consumption is thought to promote DNA damage and tumor development through the formation of cytotoxic and genotoxic compounds in the colon [172]. Elevated heme iron intake has also been positively associated with other GI malignancies, including gastric, pancreatic, and esophageal cancers [173]. In women, particularly those postmenopausal or with higher body mass index, increased heme iron intake has been linked to a greater risk of endometrial cancer [174]. Some studies further suggest a relationship between high heme consumption and lung cancer, especially in men with low antioxidant intake [175]. Beyond cancer, high heme iron intake has been consistently linked to metabolic disorders such as type 2 diabetes and gestational diabetes, likely due to iron-induced oxidative stress and damage to pancreatic β-cells [176]. Additionally, excess heme iron has been associated with an elevated risk of coronary heart disease, possibly through mechanisms involving lipid peroxidation and vascular injury [177]. These findings collectively underscore the importance of moderating heme iron intake in the diet, especially from red and processed meats, to reduce the risk of multiple non-communicable diseases.

A population-based study was conducted to explore the associations between dietary heme iron, non-heme iron, and total iron intake with metabolic syndrome and its components [178]. The findings demonstrated that higher heme iron intake, predominantly from red meat, is positively associated with metabolic syndrome and elevated triglyceride levels, likely due to heme iron’s greater bioavailability and its potential to induce oxidative stress and inflammation. In contrast, increased non-heme and total iron intakes were linked to high blood sugar levels, particularly among individuals with lower legume consumption, which may otherwise offer protective effects against impaired glucose metabolism [178]. These results emphasize the importance of differentiating between iron types when assessing their impact on metabolic health outcomes.

On the other hand, a diet rich in fruits and vegetables remains widely recognized for its protective role in chronic disease prevention [179]. Beyond their impact on iron metabolism, plant-based foods offer a wide spectrum of health benefits, supported by growing evidence linking their regular consumption to reduced risks of cardiovascular disease, cancer, stroke, cataracts, diverticulosis, chronic obstructive pulmonary disease, and hypertension. These benefits are attributed to the presence of fiber, vitamins (especially folate and vitamin C), potassium, and numerous phytochemicals such as carotenoids and flavonoids [179]. Table 4 provides an overview of key fruit and vegetable groups, highlighting their major health benefits and associated bioactive components.

**Table 4:** Overview of key fruit and vegetable groups, their associated health benefits, and protective components.

Food Group	Examples	Key Health Benefits	Protective Components/Notes	References
Dark-green leafy vegetables	Spinach, kale, collards	Cardiovascular health, cancer prevention, folic acid	High in folic acid, antioxidants (carotenoids, flavonoids)	[180]
		source		
Cruciferous vegetables	Broccoli, cauliflower, Brussels sprouts	Cancer prevention	Contain dithiothiones, indoles, isothiocyanates	[181]
Yellow-orange vegetables	Carrots, sweet potatoes, pumpkin	Eye health, cancer prevention	Rich in carotenoids (beta-carotene)	[182]
Allium vegetables	Garlic, onions, leeks	Cancer prevention, cardiovascular benefits	Sulfur-containing compounds	[180]
Citrus fruits	Oranges, grapefruits, lemons	Immune support, cancer prevention, heart health	High in vitamin C, flavonoids, folic acid	[180]
Deep-yellow-orange fruits	Mango, papaya, apricot	Antioxidant support, cancer prevention	Carotenoids, vitamin C	[181]
General fruit intake	Apples, pears, bananas, berries	Cardiovascular health, cholesterol control, fiber source	High in fiber, antioxidants	[180]
Potassium-rich vegetables/fruits	Bananas, potatoes, leafy greens	Hypertension control	Potassium content	[180,183]
Fiber-rich fruits and vegetables	Most fruits and vegetables	Cholesterol control, diverticulosis prevention	Insoluble and soluble fiber	[180]

This table highlights the role of specific plant-based foods in the prevention of chronic diseases through their micronutrient and phytochemical content.

### 5.2. Supplementation

Supplementation is an effective and cost-efficient method for treating ID over short periods, such as during pregnancy. It can be administered either orally or parenterally. Parenteral iron, which in practice is almost always administered intravenously, is recommended when oral iron is insufficient, such as in cases of significant blood loss (e.g., heavy menstrual bleeding, hereditary hemorrhagic telangiectasia), increased demand during the 2nd and 3rd trimesters of pregnancy, impaired absorption (e.g., post-bariatric surgery, IBD), or conditions with systemic inflammation and elevated hepcidin (e.g., cancer, chemotherapy-induced anemia, CKD) [184]. Extensive evidence confirms the safety and efficacy of IV iron [185,186,187]. However, oral iron remains the first-line treatment for ID as it is cost-effective, widely available, and generally sufficient for correcting mild to moderate ID.

Iron supplements on the market differ widely in their composition, dosage, bioavailability, and cost. Iron bioavailability is defined as the portion of iron in an oral supplement that is absorbed and incorporated into red blood cells [188]. Common forms of iron used in supplements are iron salts, in both ferrous and ferric forms, such as ferrous sulfate, ferrous gluconate, ferrous fumarate, and ferric citrate. The ferrous form of iron is preferred due to its higher bioavailability; ferric iron has very low solubility at near-neutral or alkaline pH and must be reduced to ferrous iron before it can be absorbed by enterocytes [189]. The bioavailability of iron from ferric iron preparations is typically 3 to 4 times lower than that of ferrous sulfate; however, they tend to have a lower incidence of side effects [188]. Different forms of iron in supplements contain varying amounts of elemental iron. For example, ferrous fumarate contains 33%, ferrous sulfate contains 20%, and ferrous gluconate contains 12% elemental iron by weight [95]. An important yet not fully explained clinical drawback of oral iron therapy is that it often causes significant GI side effects, such as constipation, stomach pain, nausea, and bloating, which frequently leads to poor patient adherence to the treatment. As many as 60% of people taking oral iron supplements report GI discomfort [188]. Such GI problems result in up to 50% of patients prescribed oral iron being non-adherent to treatment, causing their IDA to persist [190]. Although millions of patients take oral iron, the mechanisms behind the GI side effects are not fully understood. There are various theories about the causes, including the action of hydroxyl radicals, lipid peroxidation, cell damage, and changes in the microbiota [191].

The use of iron in extended-release tablets has been studied with the aim of reducing GI side effects; however, it has been found that iron absorption is significantly higher from conventional ferrous sulfate tablets. Other forms of supplemental iron are also available, such as heme iron polypeptide, Sucrosomial^®^ iron, amino acid chelated iron, and iron polysaccharide complexes, which are believed to have fewer GI side effects compared to salts [191].

The traditional recommendation for daily iron supplementation has been 150–200 mg of elemental iron, split into two or three doses [188]. However, recent studies suggest that this regimen may not be justified, as the absorption of iron from high oral doses is limited. Furthermore, unabsorbed iron can cause intestinal irritation, inflammation, and dysbiosis, which can decrease patient adherence to the treatment [190]. Research has shown that high doses of oral iron supplements increase hepcidin levels, which inhibit iron absorption for up to 24 h. It has been found that administering supplements at 48 h intervals improves iron absorption, as this allows sufficient time for hepcidin levels to drop and the mucosal absorption block to resolve [192]. Other studies have also confirmed that iron absorption improves with single daily doses or alternate-day dosing [158]. Recent studies using serum hepcidin profiles and stable iron isotopes to quantify iron absorption have also demonstrated that an oral iron dose of ≥60 mg in women with ID and ≥100 mg in women with IDA triggers an acute increase in hepcidin levels lasting 24 h, which subsides after 48 h. Even a mild increase in serum iron levels triggers hepcidin secretion, which limits iron absorption. Therefore, to enhance iron absorption, oral doses of ≥60 mg should be given on alternate days [189]. Oral iron doses of ≤40 mg do not appear to trigger an acute increase in circulating hepcidin levels in individuals with ID. Plasma hepcidin levels follow a circadian rhythm, typically increasing throughout the day. This rise is further amplified by a morning iron dose; therefore, iron should not be administered in the afternoon or evening following a morning dose. In conclusion, these studies suggest that the optimal dosing regimen is either daily oral doses of ≤40 mg or doses of ≥60 mg every other day, combined with ascorbic acid in a molar ratio of at least 2:1 (about 6 mg of ascorbic acid per 1 mg of iron). Administering these as single morning doses is recommended to maximize iron absorption in women with ID and mild IDA.

The British Society of Gastroenterology recommends starting treatment for IDA with a daily dose of 50–100 mg of elemental iron, taken as a single pill (such as iron sulfate, fumarate, or gluconate). If this is not well tolerated, options include reducing the dose to one pill every other day, trying alternative oral preparations, or considering IV iron therapy [155]. The American Gastroenterological Association also suggests using lower doses of iron supplements or adopting an every-other-day dosing schedule [193].

In areas where anemia affects 20% or more of women of reproductive age, the WHO advises intermittent supplementation with iron and folic acid. Intermittent iron supplementation refers to the consumption of iron supplements one, two, or three times a week on non-consecutive days. Randomized controlled trials have found that intermittent supplementation with iron and folic acid is equally effective as daily supplementation in treating IDA, while causing fewer side effects and improving adherence to therapy [158].

Preventive treatment with iron sulfate (60 mg for adults and 30 mg for children) is recommended in regions with a high prevalence of IDA. However, universal supplementation in countries with high rates of malaria and/or other infections is controversial, as ID may be an adaptive response that helps protect against pathogens [194].

Overall, reducing the dose of iron decreases the amount of unabsorbed iron in the intestines, thereby reducing the frequency of side effects associated with iron therapy. It appears that the best way to restore iron levels with minimal side effects is by using fewer doses of iron (even just two doses per week), which is also more cost-effective.

Oral iron should be taken on an empty stomach, at least one hour before meals, as many foods and drinks contain inhibitors of iron absorption. If this causes epigastric pain and nausea, supplements can be taken with meals to reduce side effects, although this may also decrease absorption [191]. The fraction of absorbed iron from supplements ranges from 2% to 13% when taken with food, compared to 5% to 28% when taken on an empty stomach [188]. If supplements are taken with meals, including foods or drinks rich in ascorbic acid can counteract the effects of dietary inhibitors and enhance the absorption of iron supplements.

Many multivitamin and mineral supplements contain iron, but in low doses and they often include other minerals, such as zinc, that interfere with iron absorption [191]. Iron should not be taken with antacids or proton pump inhibitors, as the increased stomach pH can reduce its solubility and absorption [74].

Patients should be monitored during the first 4 weeks to track the response of Hb to oral iron therapy and the treatment should continue for about three months after Hb levels normalize, to restore iron stores fully. Iron replacement is generally considered adequate when serum ferritin levels reach 100 µg/L [155]. The response to oral iron supplementation is an improvement in Hb levels, usually noticeable after 2–4 weeks of treatment [120]. A failure to increase Hb by at least 10 g/L after 2 weeks of daily oral iron replacement therapy is a strong predictor of an eventual inability to achieve a sustained hematological response. The effectiveness of iron supplementation can also be monitored by tracking the reticulocyte count. Reticulocytosis typically occurs 7–10 days after the initiation of iron therapy. Additionally, the reticulocyte Hb content (measured in picograms) serves as an early indicator of response to iron therapy, increasing within a few days of starting treatment [195]. Similarly, iron therapy rapidly increases serum iron levels and TSAT within 1–2 weeks.

Arranging monitoring two weeks after initiating oral iron replacement therapy may be logistically challenging in all cases. Given the lower iron doses in alternate-day regimens, a 28-day review may be more suitable. At this time, an increase in Hb level of 20 g/L or normalization is considered an adequate response. If there is no response to treatment, it is necessary to assess compliance (due to side effects or other reasons), malabsorption, or the possibility of ongoing blood loss that exceeds iron intake [155]. The ideal follow-up protocol after iron replacement therapy has yet to be determined. Once Hb returns to the normal range, checking blood counts every three months for a year, followed by every six months for 2–3 years, would be a reasonable approach. While SF is a reliable indicator of total body iron stores, there is insufficient evidence to support routine ferritin monitoring.

Supplementation often improves cognitive function and iron supplementation during pregnancy has been shown to positively impact the cognition of offspring. In adults with ID, iron therapy restores brain function, while ID in children can have irreversible harmful effects on brain development [46].

On the other hand, iron supplementation in children without ID may have adverse effects. Some studies have reported that infants and toddlers with adequate iron status who received supplements exhibited reduced weight gain and growth compared to controls [196,197]. Additionally, randomized controlled trials have shown that infants assigned to high-iron formula scored lower on cognitive assessments—such as visual memory, reading comprehension, and mathematics, compared to those who received low-iron formula [198,199]. However, the optimal dosage of iron that would prevent such effects remains uncertain, as other confounding factors may also play a role.

Iron supplements may also cause additional side effects. Zimmermann and colleagues have investigated their impact on the gut microbiome, noting that bacteria require iron to thrive. In breastmilk, the small amount of iron is bound to protective proteins such as lactoferrin, which limits its availability to pathogenic bacteria—an important defense for infants with immature immune systems. Administering iron-containing micronutrient powders to six-month-old infants, as commonly recommended, could therefore lead to adverse effects [49].

However, many clinicians and researchers agree that in cases of ID or IDA accompanied by symptoms, supplementation is often one of the quickest ways to support improvement.

While iron supplementation is essential for preventing and treating IDA, it must be approached with caution in settings where infectious diseases are prevalent. Evidence suggests that in malaria-endemic areas, particularly where healthcare infrastructure and malaria control are inadequate, high-dose oral iron given without food may increase the risk and severity of malaria and bacterial infections [200]. This is primarily due to the formation of NTBI—a labile form of circulating iron that arises when iron influx into plasma exceeds Tf-binding capacity [55]. NTBI is readily utilized by pathogens and may enhance malarial parasite sequestration in brain and gut capillaries, increasing the risk of cerebral malaria and intestinal permeability, potentially leading to bacteremia, which is commonly reported in children with severe malaria [200]. Additionally, excess iron in the gut lumen can stimulate the proliferation of pathogenic bacteria, further compounding infection risk [59]. The host’s natural defense against infection—mediated through hepcidin-induced hypoferremia—is blunted by routine iron supplementation, which may reduce its protective effect. These findings highlight the importance of carefully targeted supplementation, ideally integrated with infection screening, malaria prophylaxis, and inflammation monitoring, especially in resource-limited or malaria-endemic regions [200].

### 5.3. Fortification

Iron-fortified foods are formulated to support or enhance iron levels in individuals or populations with insufficient dietary iron intake to meet their nutritional needs. Iron fortification of food can be achieved either by adding iron compounds during processing or through biofortification via plant breeding or genetic engineering. Fortified foods fall into five main categories. The largest includes staple foods like wheat and maize flour, rice, salt, and milk, which are part of national iron fortification programs designed to increase iron intake across entire populations. These programs, often mandated by governments, benefit those at risk of deficiency but also reach individuals who already meet their iron needs. The second category consists of iron-fortified infant formulas and complementary foods, primarily consumed by infants and young children in high-income countries and affluent sectors of low and middle-income countries. The third category includes food products provided by aid agencies, such as micronutrient powders and lipid-based supplements, which target young children in low and middle-income countries. The fourth category covers voluntarily fortified foods, including breakfast cereals, chocolate drink powders, and bouillon cubes, which are marketed primarily to women, adolescents, and children. Finally, the fifth category is iron biofortification, where staple crops are enriched through conventional breeding or genetic engineering. This approach is particularly valuable for low-income households in remote areas with limited access to manufactured fortified foods [201].

Iron fortification of foods has long been challenging due to variations in iron absorption, changes in color and flavor of the food to which they are added, and the presence of absorption inhibitors. However, technical advances have led to effective iron-fortified foods that improve iron status [202]. These challenges have largely been addressed for staple foods in national fortification programs and commercially fortified foods for infants and young children. Commercially fortified infant foods have proven effective, and large-scale fortification of wheat and maize flour is well established [203]. While large-scale rice fortification technology has not yet been fully developed, the outlook is promising. A new extruded fortified kernel technology has replaced the less effective coating and dusting methods, and several countries are now introducing iron-fortified rice into public health programs and retail markets [204]. Further research is needed for iron-fortified salt and liquid milk, particularly [94,205].

Several government-led iron fortification programs have demonstrated significant success in reducing ID and IDA. Vietnam introduced iron-fortified fish sauce, a widely consumed condiment, which led to improved iron status among female factory workers [206]. In China, trials with iron-fortified soy sauce demonstrated reductions in IDA prevalence, prompting expanded implementation efforts [207]. The Philippines launched a rice fortification initiative targeting low-income households, focusing on improving the cost-effectiveness and stability of iron compounds during processing and cooking [208]. In the United States, the fortification of wheat and other cereal grains with iron has been a longstanding public health policy since the 1940s and is credited with contributing to the low rates of IDA in the general population [209]. Guatemala has implemented a successful program fortifying sugar with iron to improve the iron status of women and children, particularly in rural areas [210]. These programs highlight the importance of selecting culturally appropriate food vehicles and ensuring effective monitoring and evaluation to optimize the impact of iron fortification on public health.

A major challenge in recent years is infection-induced inflammation, which inhibits iron absorption. This is especially problematic in low and middle-income countries, where infections such as malaria, HIV, and GI diseases are common [211]. Additionally, obesity-related inflammation further complicates iron absorption and the monitoring of iron fortification programs [24]. High inflammation levels make it difficult to distinguish IDA from AI, highlighting the need for better quantification of inflammation’s impact on iron fortification. Ultimately, in low and middle-income countries with widespread inflammation, improving infection control, water quality, sanitation, and reducing obesity may have a greater impact on iron status and anemia than food fortification alone.

## 6. Recent Developments in Oral and Intravenous Therapies for Iron Deficiency

In recent years, several novel oral formulations and improved IV therapies have emerged. Oral supplements replenish iron stores but often cause GI side effects that limit adherence. Newer oral formulations aim to enhance absorption or improve tolerability, while IV therapies allow for rapid repletion, particularly when oral therapy fails or is contraindicated.

### 6.1. Oral Iron Therapies

GI side effects partly result from gastric acid interacting with ferrous iron (Fe^2+^), which is more reactive than ferric iron (Fe^3+^). Gastric acid converts Fe^2+^ to Fe^3+^, aiding absorption after Fe^3+^ is reduced back to Fe^2+^ by duodenal enzymes before uptake via DMT-1 in the small intestine [191]. This explains why Fe^2+^ supplements are typically taken on an empty stomach to avoid food buffering gastric acid. Newer iron formulations using Fe^3+^ avoid this issue, allowing administration with food, causing fewer side effects, and maintaining bioavailability even with acid-suppressing medications like proton pump inhibitors.

Ferric citrate is an oral iron formulation where ferric iron (Fe^3+^) is complexed with citrate. Initially used as a phosphate binder, it later received FDA approval for treating IDA in CKD patients not on kidney replacement therapy [157]. Clinical trials demonstrated that ferric citrate significantly increased Hb levels compared to placebo, though it was associated with slightly higher GI side effects like diarrhea and constipation. Studies showed greater Hb improvements in patients with more severe ID [212]. In CKD patients on dialysis, ferric citrate improved iron parameters (ferritin, TSAT), reduced erythropoiesis-stimulating agent and IV iron needs, and lowered serum phosphate and fibroblast growth factor 23 levels without increased adverse events [157]. Despite higher costs, ferric citrate may reduce overall treatment expenses by decreasing erythropoiesis-stimulating agent and IV iron requirements. Meta-analyses confirm its efficacy in improving iron status in CKD patients [211].

Maltol is a naturally occurring flavor enhancer that structurally resembles the iron chelator deferiprone and forms a lipophilic 3:1 complex with ferric iron (Fe^3+^). Ferric maltol facilitates iron delivery to enterocytes while keeping the unabsorbed iron in a redox-inert, chelated form. Prior to absorption, iron dissociates from the complex, allowing free maltol to be absorbed separately, metabolized, and subsequently excreted in the urine. In clinical trials (mainly in IBD and CKD patients with IDA), ferric maltol significantly raised Hb; mean Hb increases were on the order of ≥2 g/dL by 12–16 weeks of therapy. SF and TSAT levels also increased [213]. The compound was approved for medical use in the European Union in 2016 and in the United States in 2019. It has demonstrated a favorable safety profile, with GI side effects such as nausea, constipation, and diarrhea occurring at rates comparable to placebo in phase III trials. In studies involving patients with IBD, treatment discontinuation due to adverse effects was approximately 10%, similar to the placebo group [214]. Ferric maltol’s novel mechanism and trial-proven efficacy make it a promising oral alternative to traditional ferrous salts, though it is substantially more expensive.

Sucrosomial^®^ iron utilizes a phospholipid bilayer and a protective layer of sucrose esters of fatty acids—together forming a structure known as a Sucrosome^®^—to safeguard iron pyrophosphate until it reaches the intestines. The Sucrosome^®^ protects iron through the stomach and enables absorption of intact nanoparticle complexes via paracellular and transcellular routes, bypassing the usual DMT-1 transporter [215]. Clinical studies (in IBD, chronic disease, postpartum anemia, etc.) show that Sucrosomial^®^ iron improves Hb effectively, although increases in SF are often modest [95]. Importantly, Sucrosomial^®^ iron has an excellent safety profile; in one 12-week study, 96.6% of IBD patients completed therapy and only 17% reported mild GI symptoms [216]. Even patients who had not tolerated ferrous sulfate showed good adherence to Sucrosomial^®^ iron [217]. Thus, this novel formulation appears to significantly reduce GI side effects. It is sold in some countries (mostly as a medical supplement) and represents an innovative delivery system, albeit at considerably higher cost than generic iron salts.

Liposomal iron is an oral iron supplement that has gained attention in recent years due to its improved bioavailability and tolerability. Unlike traditional iron salts, liposomal iron encapsulates iron in phospholipid vesicles—called liposomes—protecting it from degradation in the GI tract and enhancing its absorption through the intestinal mucosa [218]. This delivery system enables more efficient uptake, even in individuals with inflammation or GI disorders [219]. Trials of liposomal iron in CKD and IBD patients have shown that it safely increases Hb levels; for example, liposomal iron improved Hb modestly in CKD patients, though generally less than IV iron [220]. Like Sucrosomial^®^ iron, liposomal iron is associated with significantly fewer GI side effects—such as nausea, constipation, and abdominal discomfort—which are common causes of poor adherence to conventional iron therapies [221]. Thus, liposomal iron offers another “nano-carrier” oral option, with efficacy and tolerability intermediate between ferrous salts and IV therapy.

Another novel iron formulation based on nanoparticles, a dietary ferritin analog called iron hydroxide adipate tartrate, is being investigated for its potential to treat ID in humans with fewer side effects [222]. The ACCESS trial, a double-blind, double-dummy randomized clinical study, showed that iron conjugated with N-aspartyl-casein (Fe-ASP), was as effective as traditional iron sulfate in increasing Hb levels in patients with IDA [223]. Fe-ASP also led to greater improvements in other hematological markers, such as RBC count and reticulocyte levels. Although the study did not focus on clinical symptoms or GI side effects, Fe-ASP appeared to offer better GI tolerability and absorption. While previous studies support these findings, larger trials are needed to confirm them. Strengths of the study include its real-world patient population, while limitations include a small, single-center design and a dosing schedule that may not align with current recommendations. The novel hematological response score used also requires further validation.

Research continues to explore innovative iron delivery systems, with the European Medicines Agency’s recent positive opinion on Xoanacyl^®^ (ferric citrate formulated as a coordination complex) marking a promising development. Designed for adults with CKD and coexisting ID and hyperphosphataemia, Xoanacyl^®^ offers a dual mechanism; it provides absorbable iron while simultaneously binding dietary phosphate. In the GI tract, ferric iron (210 mg per 1 g tablet) is reduced and absorbed to support Hb synthesis, whereas the unabsorbed fraction binds phosphate, lowering serum phosphorus. Clinical trials in non-dialysis CKD patients demonstrated improved Hb levels and transferrin saturation, alongside reduced phosphate levels. In dialysis-dependent patients, phosphate-lowering efficacy was comparable to that of sevelamer carbonate. Common adverse effects include diarrhea, abdominal pain, and nausea [224].

### 6.2. Intravenous Iron Therapies

Ferric carboxymaltose (FCM; Ferinject^®^, Injectafer^®^) is an IV iron formulation widely approved for the treatment of ID, with or without anemia. It consists of a colloidal solution of nanoparticles containing a polynuclear iron(III)-(oxyhydr)oxide core stabilized by carboxymaltose. A major advantage of this preparation is the ability to administer a high dose of iron—up to 750 mg in the US and 1000 mg in the EU—over a short 15 min infusion, allowing for fewer administrations compared to other IV iron products such as iron sucrose. Clinical trials and real-world data consistently demonstrate that FCM is effective and well tolerated across diverse patient populations with ID or IDA, including individuals with chronic heart failure, CKD, IBD, heavy menstrual bleeding, postpartum anemia, perioperative anemia, and ID during pregnancy [225]. Notably, the FAIR-HF and CONFIRM-HF randomized controlled trials reported significant improvements in patient global assessment, functional status, and anemia correction. FCM is generally associated with a low incidence of hypersensitivity reactions and better GI tolerability compared to oral ferrous sulfate. The most frequently observed laboratory abnormality is transient, asymptomatic hypophosphatemia. Although its acquisition cost is higher than some alternatives, this may be offset by reduced healthcare utilization, making it a cost-effective option [226]. In June 2023, Injectafer^®^ was approved for the treatment of ID in patients with NYHA class II/III heart failure to improve exercise capacity—marking the first IV iron indication specifically for heart failure [227].

Ferric derisomaltose (FDI; Monofer^®^, Monoferric^®^) is a stable iron(III)–isomaltoside complex designed for IV use, allowing administration of doses up to 1000 mg over approximately 20 min. Its efficacy in correcting IDA is comparable to that of FCM. In a randomized clinical trial involving patients with IBD, both FDI and FCM produced similar increases in Hb levels. A key differentiating factor between these formulations lies in phosphate metabolism. In the PHOSPHARE-IBD study, hypophosphatemia (defined as serum phosphate < 2.0 mg/dL) occurred in only 8.3% of patients treated with FDI, compared to 51.0% of those receiving FCM [228]. Other adverse effects—such as hypersensitivity reactions, hypotension, and GI symptoms—are uncommon and occur at rates similar to those observed with FCM. FDI is approved for use in adults with IDA in both the European Union and the United States (approved in 2020), and it is often preferred when large single-dose infusions are needed, particularly in patients at risk of hypophosphatemia. In the context of chronic heart failure, the IRONMAN trial investigated the use of FDI and reported modest clinical benefits; however, the study narrowly missed its primary endpoint of reducing hospitalizations [229].

Ferumoxytol (Feraheme^®^) is a superparamagnetic iron oxide nanoparticle initially developed as an MRI contrast agent. Its iron-replenishing effects led to its approval in the US in 2009 for the treatment of ID in patients with CKD. Due to potential MRI signal interference, radiologists should be notified if imaging is planned within 8 weeks of administration. Rapid injection (17 s) was initially used but led to increased hypersensitivity reactions—now attributed to complement activation—prompting a boxed warning and a minimum 15 min infusion time. A 2017 randomized trial comparing ferumoxytol with FCM found similar safety profiles, with FCM causing more hypophosphatemia [230]. Ferumoxytol showed no clinically significant phosphate depletion. Although the approved protocol requires two 510 mg doses, growing evidence supports a single 1020 mg infusion over 30 min, with over 2000 such cases reported without serious adverse events. A generic version has recently been approved, though preliminary reports suggest a higher rate of mild infusion reactions, warranting caution [231].

Recent research continues to advance IV iron therapies across diverse patient populations. In heart failure with reduced ejection fraction (HFrEF), IV iron has shown promising benefits. A large clinical trial presented in 2025 demonstrated that IV iron reduced the risk of first cardiovascular death or hospitalization for heart failure and improved quality of life and functional capacity, particularly in the first year of treatment [232]. These findings support current recommendations for IV iron use in HFrEF patients with ID. Additionally, a major study in India involving over 4000 pregnant participants confirmed that a single IV iron dose administered in the early second trimester is both safe and effective in correcting anemia, with improved Hb levels and minimal adverse effects [233]. These developments highlight the growing role of IV iron as a safe and efficient alternative to oral supplementation, especially in populations with poor tolerance or inadequate response to oral iron.

## 7. Conclusions

ID and IDA remain major global health challenges, affecting a substantial portion of the population, particularly vulnerable groups such as children, pregnant women, and individuals in low-resource settings. Despite growing awareness and improved understanding of iron metabolism, significant gaps persist in early diagnosis, individualized treatment, and population-level prevention strategies. The recognition that ID can impair health even in the absence of anemia underscores the importance of early detection and timely intervention. Recent advances in diagnostic methods, coupled with innovations in iron supplementation—both oral and parenteral—offer promising avenues to enhance treatment efficacy while minimizing side effects. However, a one-size-fits-all approach is no longer adequate. Personalized management that considers the underlying etiology, inflammatory status, and patient-specific factors is essential to improve outcomes and reduce the burden of this preventable condition.

## 8. Future Directions

Future research should prioritize the development and validation of accessible, affordable biomarkers for early-stage ID, especially in resource-limited settings. A key need is the precise mapping of the global and regional prevalence and severity of ID and IDA to support targeted interventions and informed policy-making. Longitudinal studies are needed to clarify the long-term effects of early-life ID on neurodevelopment and chronic disease risk. Additionally, more attention must be given to optimizing iron interventions in special populations, such as individuals with chronic inflammation or GI disorders. A more nuanced understanding of iron metabolism in the context of inflammation is needed. The presence of infections and inflammation alters iron biomarkers and limits the efficacy of interventions, particularly iron-fortified foods. Despite technical advances in iron fortification, without addressing the inflammation barrier, their impact will remain limited. A comprehensive, multisectoral strategy—integrating nutritional, clinical, and public health approaches—will be essential for reducing anemia prevalence and improving global iron health. This includes food fortification, improved dietary guidance, context-specific supplementation programs, public education on the importance of iron and other essential nutrients, and expanded access to healthcare services that enable early detection and timely treatment. Such coordinated efforts are critical not only for achieving international targets for anemia reduction but also for addressing persistent health inequities across populations.

## Data Availability

No new data were created or analyzed in this study. Data sharing is not applicable to this article.

## Abbreviations

The following abbreviations are used in this manuscript:

ID	iron deficiency
IDA	iron deficiency anemia
Hb	hemoglobin
WHO	World Health Organization
NHANES	National Health and Nutrition Examination Survey
GI	gastrointestinal
IBD	inflammatory bowel disease
SF	serum ferritin
UNICEF	United Nations International Children’s Emergency Fund
AE	anemia in the elderly
AI	anemia of inflammation
CKD	chronic kidney disease
IRIDA	iron-refractory iron deficiency anemia
YLDs	years lived with disability
ROS	reactive oxygen species
Dcytb	duodenal cytochrome b
DMT-1	divalent metal transporter 1
FPN	ferroportin
Heph	hephaestin
Tf	transferrin
sTfR	soluble transferrin receptor
TIBC	total iron-binding capacity
FBC	full blood count
MCV	mean corpuscular volume
MCH	mean corpuscular hemoglobin
MCHC	mean corpuscular hemoglobin concentration
RDW	red cell distribution width
NTBI	non-transferrin-bound iron
CRP	C-reactive protein
EHA	European Hematology Association
CDC	Centers for Disease Control and Prevention
Fe-ASP	iron conjugated with N-aspartyl-casein
IV	intravenous
FCM	ferric carboxymaltose
FDI	ferric derisomaltose
HFrEF	heart failure with reduced ejection fraction
ERFE	erythroferrone
BMP	bone morphogenetic protein
HIF-2α	hypoxia-inducible factor 2 alpha
HRI	heme-regulated inhibitor kinase
IREs	iron-responsive elements
UTRs	untranslated regions
CNN	convolutional neural networks

## References

[1] Pasricha S.R., Tye-Din J., Muckenthaler M.U., Swinkels D.W. (2021). Iron Deficiency. Lancet.

[2] Abioye A.I., Andersen C.T., Sudfeld C.R., Fawzi W.W. (2020). Anemia, Iron Status, and HIV: A Systematic Review of the Evidence. Adv. Nutr..

[3] Iron Deficiency without Anemia: Indications for Treatment–GREM–Gynecological and Reproductive Endocrinology & Metabolism. https://gremjournal.com/journal/04-2020/iron-deficiency-without-anemia-indications-for-treatment/.

[4] Zimmermann M.B., Hurrell R.F. (2007). Nutritional Iron Deficiency. Lancet.

[5] WHO/UNICEF Discussion Paper: The Extension of the 2025 Maternal, Infant and Young Child Nutrition Targets to 2030–UNICEF DATA. https://data.unicef.org/resources/who-unicef-discussion-paper-nutrition-targets/.

[6] Kassebaum N.J., Jasrasaria R., Naghavi M., Wulf S.K., Johns N., Lozano R., Regan M., Weatherall D., Chou D.P., Eisele T.P. (2014). A Systematic Analysis of Global Anemia Burden from 1990 to 2010. Blood.

[7] Gardner W.M., Razo C., McHugh T.A., Hagins H., Vilchis-Tella V.M., Hennessy C., Taylor H.J., Perumal N., Fuller K., Cercy K.M. (2023). Prevalence, Years Lived with Disability, and Trends in Anaemia Burden by Severity and Cause, 1990–2021: Findings from the Global Burden of Disease Study 2021. Lancet Haematol..

[8] Lemoine A., Tounian P. (2020). Childhood Anemia and Iron Deficiency in Sub-Saharan Africa–Risk Factors and Prevention: A Review. Arch. Pédiatr.

[9] Rakanita Y., Sinuraya R.K., Suradji E.W., Suwantika A.A., Syamsunarno M.R.A., Abdulah R. (2020). The Challenges in Eradication of Iron Deficiency Anemia in Developing Countries. Syst. Rev. Pharm..

[10] Levi M., Rosselli M., Simonetti M., Brignoli O., Cancian M., Masotti A., Pegoraro V., Cataldo N., Heiman F., Chelo M. (2016). Epidemiology of Iron Deficiency Anaemia in Four European Countries: A Population-Based Study in Primary Care. Eur. J. Haematol..

[11] Jefferds M.E.D., Mei Z., Addo Y., Hamner H.C., Perrine C.G., Flores-Ayala R., Pfeiffer C.M., Sharma A.J. (2022). Iron Deficiency in the United States: Limitations in Guidelines, Data, and Monitoring of Disparities. Am. J. Public. Health.

[12] Hercberg S., Preziosi P., Galan P. (2001). Iron Deficiency in Europe. Public. Health Nutr..

[13] Knowles J., Walters T., Yarparvar A., Brown R. (2024). A Review of Anemia Prevalence, and Prevention and Control Strategies, in the Eastern Europe and Central Asia Region. Curr. Dev. Nutr..

[14] Milman N., Taylor C.L., Merkel J., Brannon P.M. (2017). Iron Status in Pregnant Women and Women of Reproductive Age in Europe. Am. J. Clin. Nutr..

[15] Anaemia in Women and Children. https://www.who.int/data/gho/data/themes/topics/anaemia_in_women_and_children.

[16] Eussen S., Alles M., Uijterschout L., Brus F., Van Der Horst-Graat J. (2015). Iron Intake and Status of Children Aged 6–36 Months in Europe: A Systematic Review. Ann. Nutr. Metab..

[17] Tijanić I.R. (2014). Solubilni Transferenski Receptori u Dijagnostici Anemije Zbog Deficita Gvožđa. Ph.D. Thesis.

[18] Rakic L., Djokic D., Drakulovic M.B., Pejic A., Radojicic Z., Marinkovic M. (2013). Risk Factors Associated with Anemia among Serbian Non-Pregnant Women 20 to 49 Years Old. A Cross-Sectional Study. Hippokratia.

[19] Petrović V. (2020). Zdravstveno Stanje Stanovništva AP Vojvodine 2019.

[20] de Benoist B., McLean E., Egli I., Cogswell M. (2008). Worldwide Prevalence of Anaemia 1993–2005: WHO Global Database on Anaemia.

[21] Camaschella C. (2019). Iron Deficiency. Blood.

[22] Bonovas S., Fiorino G., Allocca M., Lytras T., Tsantes A., Peyrin-Biroulet L., Danese S. (2016). Intravenous versus Oral Iron for the Treatment of Anemia in Inflammatory Bowel Disease: A Systematic Review and Meta-Analysis of Randomized Controlled Trials. Medicine.

[23] Haile K., Yemane T., Tesfaye G., Wolde D., Timerga A., Haile A. (2021). Anemia and Its Association with Helicobacter Pylori Infection among Adult Dyspeptic Patients Attending Wachemo University Nigist Eleni Mohammad Memorial Referral Hospital, Southwest Ethiopia: A Cross-Sectional Study. PLoS ONE.

[24] Zhao L., Zhang X., Shen Y., Fang X., Wang Y., Wang F. (2015). Obesity and Iron Deficiency: A Quantitative Meta-Analysis. Obes. Rev..

[25] Bardou-Jacquet E., Island M.L., Jouanolle A.M., Détivaud Ĺ., Fatih N., Ropert M., Brissot E., Mosser A., Maisonneuve H., Brissot P. (2011). A Novel N491S Mutation in the Human SLC11A2 Gene Impairs Protein Trafficking and in Association with the G212V Mutation Leads to Microcytic Anemia and Liver Iron Overload. Blood Cells Mol. Dis..

[26] Sun B., Tan B., Zhang P., Zhu L., Wei H., Huang T., Li C., Yang W. (2024). Iron Deficiency Anemia: A Critical Review on Iron Absorption, Supplementation and Its Influence on Gut Microbiota. Food Funct..

[27] Althunibat O.Y., Saghir S.A.M., Aladaileh S.H., Rawadieh A. (2023). The Impact of Weight Loss Diet Programs on Anemia, Nutrient Deficiencies, and Organ Dysfunction Markers among University Female Students: A Cross-Sectional Study. Electron. J. Gen. Med..

[28] Kiss J.E., Vassallo R.R. (2018). How Do We Manage Iron Deficiency after Blood Donation?. Br. J. Haematol..

[29] Haider L.M., Schwingshackl L., Hoffmann G., Ekmekcioglu C. (2018). The Effect of Vegetarian Diets on Iron Status in Adults: A Systematic Review and Meta-Analysis. Crit. Rev. Food Sci. Nutr..

[30] Rizzo N.S., Jaceldo-Siegl K., Sabate J., Fraser G.E. (2013). Nutrient Profiles of Vegetarian and Nonvegetarian Dietary Patterns. J. Acad. Nutr. Diet..

[31] Hall N.J., Rubin G., Charnock A. (2009). Systematic Review: Adherence to a Gluten-Free Diet in Adult Patients with Coeliac Disease. Aliment. Pharmacol. Ther..

[32] Gasche C., Lomer M.C.E., Cavill I., Weiss G. (2004). Iron, Anaemia, and Inflammatory Bowel Diseases. Gut.

[33] McAfee A.J., McSorley E.M., Cuskelly G.J., Moss B.W., Wallace J.M.W., Bonham M.P., Fearon A.M. (2010). Red Meat Consumption: An Overview of the Risks and Benefits. Meat Sci..

[34] Al Hasan S.M., Hassan M., Saha S., Islam M., Billah M., Islam S. (2016). Dietary Phytate Intake Inhibits the Bioavailability of Iron and Calcium in the Diets of Pregnant Women in Rural Bangladesh: A Cross-Sectional Study. BMC Nutr..

[35] Simcox J.A., McClain D.A. (2013). Iron and Diabetes Risk. Cell Metab..

[36] Engert A., Balduini C., Brand A., Coiffier B., Cordonnier C., Döhner H., De Wit T.D., Eichinger S., Fibbe W., Green T. (2016). The European Hematology Association Roadmap for European Hematology Research: A Consensus Document. Haematologica.

[37] Bach V., Schruckmayer G., Sam I., Kemmler G., Stauder R. (2014). Prevalence and Possible Causes of Anemia in the Elderly: A Cross-Sectional Analysis of a Large European University Hospital Cohort. Clin. Interv. Aging.

[38] Girelli D., Marchi G., Camaschella C. (2018). Anemia in the Elderly. Hemasphere.

[39] Awaluddin S.M., Shahein N.A., Rahim N.C.A., Zaki N.A.M., Nasaruddin N.H., Saminathan T.A., Alias N., Ganapathy S.S., Ahmad N.A. (2021). Anemia among Men in Malaysia: A Population-Based Survey in 2019. Int. J. Environ. Res. Public Health.

[40] Rohr M., Brandenburg V., Brunner-La Rocca H.P. (2023). How to Diagnose Iron Deficiency in Chronic Disease: A Review of Current Methods and Potential Marker for the Outcome. Eur. J. Med. Res..

[41] Larson L.M., Kubes J.N., Ramírez-Luzuriaga M.J., Khishen S., H Shankar A., Prado E.L. (2019). Effects of Increased Hemoglobin on Child Growth, Development, and Disease: A Systematic Review and Meta-Analysis. Ann. N. Y. Acad. Sci..

[42] Fu X.Y., Xie X.T. (2018). Association between Iron Deficiency and Brain Developmental Disorder in Children. Zhongguo Dang Dai Er Ke Za Zhi.

[43] Hua M., Shi D., Xu W., Zhu L., Hao X., Zhu B., Shu Q., Lozoff B., Geng F., Shao J. (2023). Differentiation between Fetal and Postnatal Iron Deficiency in Altering Brain Substrates of Cognitive Control in Pre-Adolescence. BMC Med..

[44] Baron D.M., Hochrieser H., Posch M., Metnitz B., Rhodes A., Moreno R.P., Pearse R.M., Metnitz P. (2014). Preoperative Anaemia Is Associated with Poor Clinical Outcome in Non-Cardiac Surgery Patients. Br. J. Anaesth..

[45] Verdon F., Burnand B., Fallab Stubi C.L., Bonard C., Graff M., Michaud A., Bischoff T., De Vevey M., Studer J.P., Herzig L. (2003). Iron Supplementation for Unexplained Fatigue in Non-Anaemic Women: Double Blind Randomised Placebo Controlled Trial. BMJ.

[46] Abbaspour N., Hurrell R., Kelishadi R. (2014). Review on Iron and Its Importance for Human Health. J. Res. Med. Sci..

[47] Gulec S., Anderson G.J., Collins J.F. (2014). Mechanistic and Regulatory Aspects of Intestinal Iron Absorption. Am. J. Physiol. Gastrointest. Liver Physiol..

[48] Schrier S.L. (2015). So You Know How to Treat Iron Deficiency Anemia. Blood.

[49] Jaeggi T., Kortman G.A.M., Moretti D., Chassard C., Holding P., Dostal A., Boekhorst J., Timmerman H.M., Swinkels D.W., Tjalsma H. (2015). Iron Fortification Adversely Affects the Gut Microbiome, Increases Pathogen Abundance and Induces Intestinal Inflammation in Kenyan Infants. Gut.

[50] Kortman G.A.M., Raffatellu M., Swinkels D.W., Tjalsma H. (2014). Nutritional Iron Turned inside out: Intestinal Stress from a Gut Microbial Perspective. FEMS Microbiol. Rev..

[51] Swanson C.A. (2003). Iron Intake and Regulation: Implications for Iron Deficiency and Iron Overload. Alcohol.

[52] Piskin E., Cianciosi D., Gulec S., Tomas M., Capanoglu E. (2022). Iron Absorption: Factors, Limitations, and Improvement Methods. ACS Omega.

[53] Ganz T. (2005). Cellular Iron: Ferroportin Is the Only Way Out. Cell Metab..

[54] Anderson G.J., Vulpe C.D. (2009). Mammalian Iron Transport. Cell. Mol. Life Sci..

[55] Brissot P., Ropert M., Le Lan C., Loréal O. (2012). Non-Transferrin Bound Iron: A Key Role in Iron Overload and Iron Toxicity. Biochim. Biophys. Acta (BBA)—Gen. Subj..

[56] Waldvogel-Abramowski S., Waeber G., Gassner C., Buser A., Frey B.M., Favrat B., Tissot J.D. (2014). Physiology of Iron Metabolism. Transfus. Med. Hemother..

[57] Sharp P., Srai S.K. (2007). Molecular Mechanisms Involved in Intestinal Iron Absorption. World J. Gastroenterol..

[58] Nemeth E., Ganz T. (2006). Regulation of Iron Metabolism by Hepcidin. Annu. Rev. Nutr..

[59] Murdoch C.C., Skaar E.P. (2022). Nutritional Immunity: The Battle for Nutrient Metals at the Host–Pathogen Interface. Nat. Rev. Microbiol..

[60] Hood M.I., Skaar E.P. (2012). Nutritional Immunity: Transition Metals at the Pathogen-Host Interface. Nat. Rev. Microbiol..

[61] Aschemeyer S., Qiao B., Stefanova D., Valore E.V., Sek A.C., Alex Ruwe T., Vieth K.R., Jung G., Casu C., Rivella S. (2018). Structure-Function Analysis of Ferroportin Defines the Binding Site and an Alternative Mechanism of Action of Hepcidin. Blood.

[62] Kautz L., Meynard D., Monnier A., Darnaud V., Bouvet R., Wang R.H., Deng C., Vaulont S., Mosser J., Coppin H. (2008). Iron Regulates Phosphorylation of Smad1/5/8 and Gene Expression of Bmp6, Smad7, Id1, and Atoh8 in the Mouse Liver. Blood.

[63] Silvestri L., Pagani A., Nai A., De Domenico I., Kaplan J., Camaschella C. (2008). The Serine Protease Matriptase-2 (TMPRSS6) Inhibits Hepcidin Activation by Cleaving Membrane Hemojuvelin. Cell Metab..

[64] Pasricha S.R., Lim P.J., Duarte T.L., Casu C., Oosterhuis D., Mleczko-Sanecka K., Suciu M., Da Silva A.R., Al-Hourani K., Arezes J. (2017). Hepcidin Is Regulated by Promoter-Associated Histone Acetylation and HDAC3. Nat. Commun..

[65] Kautz L., Jung G., Valore E.V., Rivella S., Nemeth E., Ganz T. (2014). Identification of Erythroferrone as an Erythroid Regulator of Iron Metabolism. Nat. Genet..

[66] Mastrogiannaki M., Matak P., Peyssonnaux C. (2013). The Gut in Iron Homeostasis: Role of HIF-2 under Normal and Pathological Conditions. Blood.

[67] Zhang D.L., Wu J., Shah B.N., Greutélaers K.C., Ghosh M.C., Ollivierre H., Su X.Z., Thuma P.E., Bedu-Addo G., Mockenhaupt F.P. (2018). Erythrocytic Ferroportin Reduces Intracellular Iron Accumulation, Hemolysis, and Malaria Risk. Science.

[68] Zhang D.L., Ghosh M.C., Rouault T.A. (2014). The Physiological Functions of Iron Regulatory Proteins in Iron Homeostasis–an Update. Front. Pharmacol..

[69] Bayeva M., Khechaduri A., Puig S., Chang H.C., Patial S., Blackshear P.J., Ardehali H. (2012). MTOR Regulates Cellular Iron Homeostasis through Tristetraprolin. Cell Metab..

[70] Mancias J.D., Wang X., Gygi S.P., Harper J.W., Kimmelman A.C. (2014). Quantitative Proteomics Identifies NCOA4 as the Cargo Receptor Mediating Ferritinophagy. Nature.

[71] Richardson C.L., Delehanty L.L., Bullock G.C., Rival C.M., Tung K.S., Kimpel D.L., Gardenghi S., Rivella S., Goldfarb A.N. (2013). Isocitrate Ameliorates Anemia by Suppressing the Erythroid Iron Restriction Response. J. Clin. Investig..

[72] Nai A., Lidonnici M.R., Rausa M., Mandelli G., Pagani A., Silvestri L., Ferrari G., Camaschella C. (2015). The Second Transferrin Receptor Regulates Red Blood Cell Production in Mice. Blood.

[73] Sonnweber T., Nachbaur D., Schroll A., Nairz M., Seifert M., Demetz E., Haschka D., Mitterstiller A.M., Kleinsasser A., Burtscher M. (2014). Hypoxia Induced Downregulation of Hepcidin Is Mediated by Platelet Derived Growth Factor BB. Gut.

[74] Hurrell R., Egli I. (2010). Iron Bioavailability and Dietary Reference Values. Am. J. Clin. Nutr..

[75] Gibson R.S., Bailey K.B., Gibbs M., Ferguson E.L. (2010). A Review of Phytate, Iron, Zinc, and Calcium Concentrations in Plant-Based Complementary Foods Used in Low-Income Countries and Implications for Bioavailability. Food Nutr. Bull..

[76] Cook J.D., Dassenko S.A., Lynch S.R. (1991). Assessment of the Role of Nonheme-Iron Availability in Iron Balance. Am. J. Clin. Nutr..

[77] Hoppe M., Ross A.B., Svelander C., Sandberg A.S., Hulthén L. (2019). Low-Phytate Wholegrain Bread Instead of High-Phytate Wholegrain Bread in a Total Diet Context Did Not Improve Iron Status of Healthy Swedish Females: A 12-Week, Randomized, Parallel-Design Intervention Study. Eur. J. Nutr..

[78] Armah S.M., Boy E., Chen D., Candal P., Reddy M.B. (2015). Regular Consumption of a High-Phytate Diet Reduces the Inhibitory Effect of Phytate on Nonheme-Iron Absorption in Women with Suboptimal Iron Stores. J. Nutr..

[79] Rana A., Samtiya M., Dhewa T., Mishra V., Aluko R.E. (2022). Health Benefits of Polyphenols: A Concise Review. J. Food Biochem..

[80] Lesjak M., Balesaria S., Skinner V., Debnam E.S., Srai S.K.S. (2019). Quercetin Inhibits Intestinal Non-Haem Iron Absorption by Regulating Iron Metabolism Genes in the Tissues. Eur. J. Nutr..

[81] Lesjak M., Hoque R., Balesaria S., Skinner V., Debnam E.S., Srai S.K.S., Sharp P.A. (2014). Quercetin Inhibits Intestinal Iron Absorption and Ferroportin Transporter Expression In Vivo and In Vitro. PLoS ONE.

[82] Dijiong W., Xiaowen W., Linlong X., Wenbin L., Huijin H., Baodong Y., Yuhong Z. (2019). Iron Chelation Effect of Curcumin and Baicalein on Aplastic Anemia Mouse Model with Iron Overload. Iran. J. Basic. Med. Sci..

[83] Zhen A.W., Nguyen N.H., Gibert Y., Motola S., Buckett P., Wessling-Resnick M., Fraenkel E., Fraenkel P.G. (2013). The Small Molecule, Genistein, Increases Hepcidin Expression in Human Hepatocytes. Hepatology.

[84] Patchen B., Koppe T., Cheng A., Seo Y.A., Wessling-Resnick M., Fraenkel P.G. (2016). Dietary Supplementation with Ipriflavone Decreases Hepatic Iron Stores in Wild Type Mice. Blood Cells Mol. Dis..

[85] Mu M., An P., Wu Q., Shen X., Shao D., Wang H., Zhang Y., Zhang S., Yao H., Min J. (2016). The Dietary Flavonoid Myricetin Regulates Iron Homeostasis by Suppressing Hepcidin Expression. J. Nutr. Biochem..

[86] Bayele H.K., Balesaria S., Srai S.K.S. (2015). Phytoestrogens Modulate Hepcidin Expression by Nrf2: Implications for Dietary Control of Iron Absorption. Free Radic. Biol. Med..

[87] Vanhees K., Godschalk R.W., Sanders A., Van Waalwijk van Doorn-Khosrovani S.B., Van Schooten F.J. (2011). Maternal Quercetin Intake during Pregnancy Results in an Adapted Iron Homeostasis at Adulthood. Toxicology.

[88] Hart J.J., Tako E., Kochian L.V., Glahn R.P. (2015). Identification of Black Bean (*Phaseolus vulgaris* L.) Polyphenols That Inhibit and Promote Iron Uptake by Caco-2 Cells. J. Agric. Food Chem..

[89] Lesjak M., Srai S.K.S. (2019). Role of Dietary Flavonoids in Iron Homeostasis. Pharmaceuticals.

[90] Ma Q., Kim E.Y., Lindsay E.A., Han O. (2011). Bioactive Dietary Polyphenols Inhibit Heme Iron Absorption in a Dose-Dependent Manner in Human Intestinal Caco-2 Cells. J. Food Sci..

[91] Hallberg L., Rossander-Hulthèn L., Brune M., Gleerup A. (1993). Inhibition of Haem-Iron Absorption in Man by Calcium. Br. J. Nutr..

[92] Roughead Z.K., Zito C.A., Hunt J.R. (2005). Inhibitory Effects of Dietary Calcium on the Initial Uptake and Subsequent Retention of Heme and Nonheme Iron in Humans: Comparisons Using an Intestinal Lavage Method. Am. J. Clin. Nutr..

[93] Gaitán D., Flores S., Saavedra P., Miranda C., Olivares M., Arredondo M., de Romaña D.L., Lönnerdal B., Pizarro F. (2011). Calcium Does Not Inhibit the Absorption of 5 Milligrams of Nonheme or Heme Iron at Doses Less Than 800 Milligrams in Nonpregnant Women. J. Nutr..

[94] Henare S.J., Nur Singh N., Ellis A.M., Moughan P.J., Thompson A.K., Walczyk T. (2019). Iron Bioavailability of a Casein-Based Iron Fortificant Compared with That of Ferrous Sulfate in Whole Milk: A Randomized Trial with a Crossover Design in Adult Women. Am. J. Clin. Nutr..

[95] Cámara-Martos F., Amaro-López M.A. (2002). Influence of Dietary Factors on Calcium Bioavailability: A Brief Review. Biol. Trace Elem. Res..

[96] Hurrell R.F., Lynch S.R., Trinidad T.P., Dassenko S.A., Cook J.D. (1988). Iron Absorption in Humans: Bovine Serum Albumin Compared with Beef Muscle and Egg White. Am. J. Clin. Nutr..

[97] Awoniyi A., Daniel O., Babatunde O., Awoniyi A., Daniel O., Babatunde O. (2024). Dietary Iron Uptake and Absorption. Metabolism Annual Volume 2024.

[98] Khoja K.K., Aslam M.F., Sharp P.A., Latunde-Dada G.O. (2021). In Vitro Bioaccessibility and Bioavailability of Iron from Fenugreek, Baobab and Moringa. Food Chem..

[99] He W., Li X., Ding K., Li Y., Li W. (2019). Ascorbic Acid Can Reverse the Inhibition of Phytic Acid, Sodium Oxalate and Sodium Silicate on Iron Absorption in Caco-2 Cells. Int. J. Vitam. Nutr. Res..

[100] Villaño D., Vilaplana C., Medina S., Algaba-Chueca F., Cejuela-Anta R., Martínez-Sanz J.M., Ferreres F., Gil-Izquierdo A. (2016). Relationship between the Ingestion of a Polyphenol-Rich Drink, Hepcidin Hormone, and Long-Term Training. Molecules.

[101] Mao X., Yao G. (1992). Effect of Vitamin C Supplementations on Iron Deficiency Anemia in Chinese Children. Biomed. Environ. Sci..

[102] Teucher B., Olivares M., Cori H. (2004). Enhancers of Iron Absorption: Ascorbic Acid and Other Organic Acids. Int. J. Vitam. Nutr. Res..

[103] Cook J.D., Monsen E.R. (1977). Vitamin C, the Common Cold, and Iron Absorption. Am. J. Clin. Nutr..

[104] Cook J.D., Reddy M.B. (2001). Effect of Ascorbic Acid Intake on Nonheme-Iron Absorption from a Complete Diet. Am. J. Clin. Nutr..

[105] Layrisse M., Martínez-Torres C., Roche M. (1968). Effect of Interaction of Various Foods on Iron Absorption. Am. J. Clin. Nutr..

[106] Bœch S.B., Hansen M., Bukhave K., Jensen M., Sørensen S.S., Kristensen L., Purslow P.P., Skibsted L.H., Sandström B. (2003). Nonheme-Iron Absorption from a Phytate-Rich Meal Is Increased by the Addition of Small Amounts of Pork Meat. Am. J. Clin. Nutr..

[107] Navas-Carretero S., Pérez-Granados A.M., Sarriá B., Vaquero M.P., Carbajal A., Pedrosa M.M., Roe M.A., Fairweather-Tait S.J. (2008). Oily Fish Increases Iron Bioavailability of a Phytate Rich Meal in Young Iron Deficient Women. J. Am. Coll. Nutr..

[108] O’Flaherty E.A.A., Tsermoula P., O’Neill E.E., O’Brien N.M. (2019). Co-Products of Beef Processing Enhance Non-Haem Iron Absorption in an in Vitro Digestion/Caco-2 Cell Model. Int. J. Food Sci. Technol..

[109] Hurrell R.F., Reddy M.B., Juillerat M., Cook J.D. (2006). Meat Protein Fractions Enhance Nonheme Iron Absorption in Humans. J. Nutr..

[110] Armah C.N., Sharp P., Mellon F.A., Pariagh S., Lund E.K., Dainty J.R., Teucher B., Fairweather-Tait S.J. (2008). L-α-Glycerophosphocholine Contributes to Meat’s Enhancement of Nonheme Iron Absorption. J. Nutr..

[111] Gibson G.R., Hutkins R., Sanders M.E., Prescott S.L., Reimer R.A., Salminen S.J., Scott K., Stanton C., Swanson K.S., Cani P.D. (2017). Expert Consensus Document: The International Scientific Association for Probiotics and Prebiotics (ISAPP) Consensus Statement on the Definition and Scope of Prebiotics. Nat. Rev. Gastroenterol. Hepatol..

[112] Jeroense F.M.D., Michel L., Zeder C., Herter-Aeberli I., Zimmermann M.B. (2019). Consumption of Galacto-Oligosaccharides Increases Iron Absorption from Ferrous Fumarate: A Stable Iron Isotope Study in Iron-Depleted Young Women. J. Nutr..

[113] Giorgetti A., Husmann F.M.D., Zeder C., Herter-Aeberli I., Zimmermann M.B. (2022). Prebiotic Galacto-Oligosaccharides and Fructo-Oligosaccharides, but Not Acacia Gum, Increase Iron Absorption from a Single High-Dose Ferrous Fumarate Supplement in Iron-Depleted Women. J. Nutr..

[114] Ahmad A.M.R., Ahmed W., Iqbal S., Javed M., Rashid S. (2021). Iahtisham-ul-Haq Prebiotics and Iron Bioavailability? Unveiling the Hidden Association–A Review. Trends Food Sci. Technol..

[115] Yeung C.K., Glahn R.P., Welch R.M., Miller D.D. (2005). Prebiotics and Iron Bioavailability—Is There a Connection?. J. Food Sci..

[116] Husmann F.M.D., Zimmermann M.B., Herter-Aeberli I. (2022). The Effect of Prebiotics on Human Iron Absorption: A Review. Adv. Nutr..

[117] Christides T., Sharp P. (2013). Sugars Increase Non-Heme Iron Bioavailability in Human Epithelial Intestinal and Liver Cells. PLoS ONE.

[118] WHO (2011). Haemoglobin Concentrations for the Diagnosis of Anaemia and Assessment of Severity. Vitamin and Mineral Nutrition Information System.

[119] Goddard A.F., James M.W., McIntyre A.S., Scott B.B. (2011). Guidelines for the Management of Iron Deficiency Anaemia. Gut.

[120] Hastka J., Lasserre J.J., Schwarzbeck A., Reiter A., Hehlmann R. (1996). Laboratory Tests of Iron Status: Correlation or Common Sense?. Clin. Chem..

[121] Guyatt G.H., Oxman A.D., Ali M., Willan A., McIlroy W., Patterson C. (1992). Laboratory Diagnosis of Iron-Deficiency Anemia–An Overview. J. Gen. Intern. Med..

[122] World Health Organization (2011). Serum Ferritin Concentrations for the Assessment of Iron Status and Iron Deficiency in Populations.

[123] Sofiantin N., Kurniawan L.B., Arif M. (2021). Analysis of Ferritin Levels, TIBC and Fe Serum In Central Obesity And Non Central Obesity. Str. J. Ilm. Kesehat..

[124] Khan A., Khan W.M., Ayub M., Humayun M., Haroon M. (2016). Ferritin Is a Marker of Inflammation Rather than Iron Deficiency in Overweight and Obese People. J. Obes..

[125] Coenen J.L.L.M., Van Dieijen-Visser M.P., Van Pelt J., Van Deursen C.T.B.M., Fickers M.M.F., Van Wersch J.W.J., Brombacher P.J. (1991). Measurements of Serum Ferritin Used to Predict Concentrations of Iron in Bone Marrow in Anemia of Chronic Disease. Clin. Chem..

[126] Pfeiffer C.M., Looker A.C. (2017). Laboratory Methodologies for Indicators of Iron Status: Strengths, Limitations, and Analytical Challenges. Am. J. Clin. Nutr..

[127] Auerbach M., Adamson J.W. (2016). How We Diagnose and Treat Iron Deficiency Anemia. Am. J. Hematol..

[128] Fletcher A., Forbes A., Svenson N., Wayne Thomas D. (2022). Guideline for the Laboratory Diagnosis of Iron Deficiency in Adults (Excluding Pregnancy) and Children. J. Haematol..

[129] Wish J.B. (2006). Assessing Iron Status: Beyond Serum Ferritin and Transferrin Saturation. J. Am. Soc. Nephrol..

[130] Cook J.D. (2005). Diagnosis and Management of Iron-Deficiency Anaemia. Best Practice & Research Clinical Haematology.

[131] Daude S., Remen T., Chateau T., Danese S., Gastin I., Baumann C., Gueant J.L., Peyrin-Biroulet L. (2020). Comparative Accuracy of Ferritin, Transferrin Saturation and Soluble Transferrin Receptor for the Diagnosis of Iron Deficiency in Inflammatory Bowel Disease. Aliment. Pharmacol Ther..

[132] World Health Organization (2007). Assessing the Iron Status of Populations.

[133] Graham E.A., Felgenhauer J., Detter J.C., Labbe R.F. (1996). Elevated Zinc Protoporphyrin Associated with Thalassemia Trait and Hemoglobin E. J. Pediatr..

[134] Asobayire F.S., Adou P., Davidsson L., Cook J.D., Hurrell R.F. (2001). Prevalence of Iron Deficiency with and without Concurrent Anemia in Population Groups with High Prevalences of Malaria and Other Infections: A Study in Côte d’Ivoire. Am. J. Clin. Nutr..

[135] Choi J.W. (2005). Sensitivity, Specificity, and Predictive Value of Serum Soluble Transferrin Receptor at Different Stages of Iron Deficiency. Ann. Clin. Lab. Sci..

[136] Shin D.H., Kim H.S., Park M.J., Suh I.B., Shin K.S. (2015). Utility of Access Soluble Transferrin Receptor (sTfR) and sTfR/log Ferritin Index in Diagnosing Iron Deficiency Anemia. Ann. Clin. Lab. Sci..

[137] Cook J.D., Flowers C.H., Skikne B.S. (2003). The Quantitative Assessment of Body Iron. Blood.

[138] Bermejo F., García-López S. (2009). A Guide to Diagnosis of Iron Deficiency and Iron Deficiency Anemia in Digestive Diseases. World J. Gastroenterol..

[139] Sultana G.S., Haque S.A., Sultana T., An A. (2013). Value of Red Cell Distribution Width (RDW) and RBC Indices in the Detection of Iron Deficiency Anemia. Mymensingh Med. J..

[140] Cheng P.P., Jiao X.Y., Wang X.H., Lin J.H., Cai Y.M. (2011). Hepcidin Expression in Anemia of Chronic Disease and Concomitant Iron-Deficiency Anemia. Clin. Exp. Med..

[141] Ganz T., Nemeth E. (2009). Iron Sequestration and Anemia of Inflammation. Semin. Hematol..

[142] Kroot J.J.C., Hendriks J.C.M., Laarakkers C.M.M., Klaver S.M., Kemna E.H.J.M., Tjalsma H., Swinkels D.W. (2009). (Pre)Analytical Imprecision, between-Subject Variability, and Daily Variations in Serum and Urine Hepcidin: Implications for Clinical Studies. Anal. Biochem..

[143] Zheng X., Chen X., Jian N., Chen J., Hu P., Jiang J. (2015). A Rapid and Sensitive LC–MS–MS Method for Determination of Hepcidin-25 in Human Serum, and Measurement of Its Diurnal Rhythm for Healthy Subjects. Chromatographia.

[144] Young M.F., Glahn R.P., Ariza-Nieto M., Inglis J., Olbina G., Westerman M., O’Brien K.O. (2009). Serum Hepcidin Is Significantly Associated with Iron Absorption from Food and Supplemental Sources in Healthy Young Women. Am. J. Clin. Nutr..

[145] Murphy A.T., Witcher D.R., Luan P., Wroblewski V.J. (2007). Quantitation of Hepcidin from Human and Mouse Serum Using Liquid Chromatography Tandem Mass Spectrometry. Blood.

[146] Girelli D., Nemeth E., Swinkels D.W. (2016). Hepcidin in the Diagnosis of Iron Disorders. Blood.

[147] Weiss G., Ganz T., Goodnough L.T. (2019). Anemia of Inflammation. Blood.

[148] Jelkmann W. (2011). Regulation of Erythropoietin Production. J. Physiol..

[149] Seekircher L., Siller A., Amato M., Tschiderer L., Balog A., Astl M., Schennach H., Willeit P. (2024). HemoCue Hb-801 Provides More Accurate Hemoglobin Assessment in Blood Donors Than OrSense NBM-200. Transfus. Med. Rev..

[150] Asare J.W., Appiahene P., Donkoh E.T., Dimauro G. (2023). Iron Deficiency Anemia Detection Using Machine Learning Models: A Comparative Study of Fingernails, Palm and Conjunctiva of the Eye Images. Eng. Rep..

[151] WHO (2020). WHO Guideline on Use of Ferritin Concentrations to Assess Iron Status in Individuals and Populations.

[152] Iolascon A., Andolfo I., Russo R., Sanchez M., Busti F., Swinkels D., Aguilar Martinez P., Bou-Fakhredin R., Muckenthaler M.U., Unal S. (2024). Recommendations for Diagnosis, Treatment, and Prevention of Iron Deficiency and Iron Deficiency Anemia. Hemasphere.

[153] Bouri S., Martin J. (2018). Investigation of Iron Deficiency Anaemia. Clin. Med..

[154] Dahlerup J.F., Eivindson M., Jacobsen B.A., Jensen N.M., Jørgensen S.P., Laursen S.B., Rasmussen M., Nathan T. (2015). Diagnosis and Treatment of Unexplained Anemia with Iron Deficiency without Overt Bleeding. Dan. Med. J..

[155] Snook J., Bhala N., Beales I.L.P., Cannings D., Kightley C., Logan R.P.H., Pritchard D.M., Sidhu R., Surgenor S., Thomas W. (2021). British Society of Gastroenterology Guidelines for the Management of Iron Deficiency Anaemia in Adults. Gut.

[156] Fernández-Gaxiola A.C., De-Regil L.M. (2019). Intermittent Iron Supplementation for Reducing Anaemia and Its Associated Impairments in Adolescent and Adult Menstruating Women. Cochrane Database Syst. Rev..

[157] Pantopoulos K. (2024). Oral Iron Supplementation: New Formulations, Old Questions. Haematologica.

[158] Melina V., Craig W., Levin S. (2016). Position of the Academy of Nutrition and Dietetics: Vegetarian Diets. J. Acad. Nutr. Diet..

[159] Thane C.W., Walmsley C.M., Bates C.J., Prentice A., Cole T.J. (2000). Risk Factors for Poor Iron Status in British Toddlers: Further Analysis of Data from the National Diet and Nutrition Survey of Children Aged 1.5–4.5 Years. Public. Health Nutr..

[160] Singh A., Bains K., Kaur H. (2016). Effect of Inclusion of Key Foods on in Vitro Iron Bioaccessibility in Composite Meals. J. Food Sci. Technol..

[161] Saunders A.V., Craig W.J., Baines S.K., Posen J.S. (2013). Iron and Vegetarian Diets. Med. J. Aust..

[162] Food Sources of Iron|Dietary Guidelines for Americans. https://www.dietaryguidelines.gov/.

[163] Iron-Rich Food|List of Meats And Vegetables|Red Cross Blood. https://www.redcrossblood.org/donate-blood/blood-donation-process/before-during-after/iron-blood-donation/iron-rich-foods.html.

[164] Kraemer K., Zimmermann M.B. (2007). Nutritional Anemia.

[165] Acosta A., Amar M., Cornbluth-Szarfarc S.C. (1984). Iron Absorption from Typical Latin American Diets. Am. J. Clin. Nutr..

[166] Okwuonu I.C., Narayanan N.N., Egesi C.N., Taylor N.J. (2021). Opportunities and Challenges for Biofortification of Cassava to Address Iron and Zinc Deficiency in Nigeria. Glob. Food Sec..

[167] Rahman S., Shaheen N. (2022). Phytate-iron Molar Ratio and Bioavailability of Iron in Bangladesh. Trop. Med. Int. Health.

[168] World Health Organization Anaemia Action Alliance. https://www.who.int/teams/nutrition-and-food-safety/anaemia-action-alliance.

[169] WHO (2017). Nutritional Anaemias: Tools for Effective Prevention.

[170] World Health Organization Anaemia: Fact Sheet. https://www.who.int/news-room/fact-sheets/detail/anaemia.

[171] Hooda J., Shah A., Zhang L. (2014). Heme, an Essential Nutrient from Dietary Proteins, Critically Impacts Diverse Physiological and Pathological Processes. Nutrients.

[172] Qiao L., Feng Y. (2013). Intakes of Heme Iron and Zinc and Colorectal Cancer Incidence: A Meta-Analysis of Prospective Studies. Cancer Causes Control.

[173] Lunn J.C., Kuhnle G., Mai V., Frankenfeld C., Shuker D.E.G., Glen R.C., Goodman J.M., Pollock J.R.A., Bingham S.A. (2007). The Effect of Haem in Red and Processed Meat on the Endogenous Formation of N -Nitroso Compounds in the Upper Gastrointestinal Tract. Carcinogenesis.

[174] Matias-Guiu X., Prat J. (2013). Molecular Pathology of Endometrial Carcinoma. Histopathology.

[175] Nobles C.L., Green S.I., Maresso A.W. (2013). A Product of Heme Catabolism Modulates Bacterial Function and Survival. PLoS Pathog..

[176] Zhao Z., Li S., Liu G., Yan F., Ma X., Huang Z., Tian H. (2012). Body Iron Stores and Heme-Iron Intake in Relation to Risk of Type 2 Diabetes: A Systematic Review and Meta-Analysis. PLoS ONE.

[177] Tzonou A., Lagiou P., Trichopoulou A., Tsoutsos V., Trichopoulos D. (1998). Dietary Iron and Coronary Heart Disease Risk: A Study from Greece. Am. J. Epidemiol..

[178] Vieira D.A.D.S., Sales C.H., Cesar C.L.G., Marchioni D.M., Fisberg R.M. (2018). Influence of Haem, Non-Haem, and Total Iron Intake on Metabolic Syndrome and Its Components: A Population-Based Study. Nutrients.

[179] Van Duyn M.A.S., Pivonka E. (2000). Overview of the Health Benefits of Fruit and Vegetable Consumption for the Dietetics Professional: Selected Literature. J. Am. Diet. Assoc..

[180] Klerk M., Jansen M.C.J.F., van ‘t Veer P., Kok F.J. (1998). Fruits and Vegetables in Chronic Disease Prevention.

[181] Steinmetz K.A., Potter J.D. (1996). Vegetables, Fruit, and Cancer Prevention: A Review. J. Am. Diet. Assoc..

[182] Jacques P.F., Chylack L.T. (1991). Epidemiologic Evidence of a Role for the Antioxidant Vitamins and Carotenoids in Cataract Prevention. Am. J. Clin. Nutr..

[183] Appel L.J., Moore T.J., Obarzanek E., Vollmer W.M., Svetkey L.P., Sacks F.M., Bray G.A., Vogt T.M., Cutler J.A., Windhauser M.M. (1997). A Clinical Trial of the Effects of Dietary Patterns on Blood Pressure. N. Engl. J. Med..

[184] Van Doren L., Steinheiser M., Boykin K., Taylor K.J., Menendez M., Auerbach M. (2024). Expert Consensus Guidelines: Intravenous Iron Uses, Formulations, Administration, and Management of Reactions. Am. J. Hematol..

[185] Auerbach M., Macdougall I. (2017). The Available Intravenous Iron Formulations: History, Efficacy, and Toxicology. Hemodial. Int..

[186] Stojanovic S., Graudins L.V., Aung A.K., Grannell L., Hew M., Zubrinich C. (2021). Safety of Intravenous Iron Following Infusion Reactions. J. Allergy Clin. Immunol. Pract..

[187] Blumenstein I., Shanbhag S., Langguth P., Kalra P.A., Zoller H., Lim W. (2021). Newer Formulations of Intravenous Iron: A Review of Their Chemistry and Key Safety Aspects–Hypersensitivity, Hypophosphatemia, and Cardiovascular Safety. Expert. Opin. Drug Saf..

[188] Wu T.W., Tsai F.P. (2016). Comparison of the Therapeutic Effects and Side Effects of Oral Iron Supplements in Iron Deficiency Anemia. Drug Res..

[189] Stoffel N.U., von Siebenthal H.K., Moretti D., Zimmermann M.B. (2020). Oral Iron Supplementation in Iron-Deficient Women: How Much and How Often?. Mol. Asp. Med..

[190] Kolars B., Minakovic I., Grabovac B., Zivanovic D., Jovin V.M. (2024). Treatment Adherence and the Contemporary Approach to Treating Type 2 Diabetes Mellitus. Biomed. Pap..

[191] Tolkien Z., Stecher L., Mander A.P., Pereira D.I.A., Powell J.J. (2015). Ferrous Sulfate Supplementation Causes Significant Gastrointestinal Side-Effects in Adults: A Systematic Review and Meta-Analysis. PLoS ONE.

[192] Moretti D., Goede J.S., Zeder C., Jiskra M., Chatzinakou V., Tjalsma H., Melse-Boonstra A., Brittenham G., Swinkels D.W., Zimmermann M.B. (2015). Oral Iron Supplements Increase Hepcidin and Decrease Iron Absorption from Daily or Twice-Daily Doses in Iron-Depleted Young Women. Blood.

[193] Sonoda K. (2021). Iron Deficiency Anemia: Guidelines from the American Gastroenterological Association. Am. Fam. Physician.

[194] Clark M.A., Goheen M.M., Fulford A., Prentice A.M., Elnagheeb M.A., Patel J., Fisher N., Taylor S.M., Kasthuri R.S., Cerami C. (2014). Host Iron Status and Iron Supplementation Mediate Susceptibility to Erythrocytic Stage *Plasmodium falciparum*. Nat. Commun..

[195] Arcangelo V., Peterson A. (2006). Pharmacotherapeutics for Advanced Practice: A Practical Approach.

[196] Idjradinata P., Watkins W.E., Pollitt E. (1994). Adverse Effect of Iron Supplementation on Weight Gain of Iron-Replete Young Children. Lancet.

[197] Majumdar I., Paul P., Talib V.H., Ranga S. (2003). The Effect of Iron Therapy on the Growth of Iron-Replete and Iron-Deplete Children. J. Trop. Pediatr..

[198] Gahagan S., Delker E., Blanco E., Burrows R., Lozoff B. (2019). Randomized Controlled Trial of Iron-Fortified versus Low-Iron Infant Formula: Developmental Outcomes at 16 Years. J. Pediatr..

[199] Lozoff B., Castillo M., Clark K.M., Smith J.B. (2012). Iron-Fortified vs Low-Iron Infant Formula: Developmental Outcome at 10 Years. Arch. Pediatr. Adolesc. Med..

[200] Hurrell R.F. (2011). Safety and Efficacy of Iron Supplements in Malaria-Endemic Areas. Ann. Nutr. Metab..

[201] Hotz C., McClafferty B. (2007). From Harvest to Health: Challenges for Developing Biofortified Staple Foods and Determining Their Impact on Micronutrient Status. Food Nutr. Bull..

[202] Hurrell R.F. (2002). Fortification: Overcoming Technical and Practical Barriers. J. Nutr..

[203] Klassen-Wigger P., Barclay D.V. (2018). Market-Driven Fortification. Food Fortification in a Globalized World.

[204] Hackl L., Cercamondi C.I., Zeder C., Wild D., Adelmann H., Zimmermann M.B., Moretti D. (2016). Cofortification of Ferric Pyrophosphate and Citric Acid/Trisodium Citrate into Extruded Rice Grains Doubles Iron Bioavailability through in Situ Generation of Soluble Ferric Pyrophosphate Citrate Complexes. Am. J. Clin. Nutr..

[205] Diosady L.L., Mannar M.G.V., Krishnaswamy K. (2019). Improving the Lives of Millions through New Double Fortification of Salt Technology. Matern. Child. Nutr..

[206] Van Thuy P., Berger J., Davidsson L., Khan N.C., Lam N.T., Cook J.D., Hurrell R.F., Khoi H.H. (2003). Regular Consumption of NaFeEDTA-Fortified Fish Sauce Improves Iron Status and Reduces the Prevalence of Anemia in Anemic Vietnamese Women. Am. J. Clin. Nutr..

[207] Huo J.S., Yin J.Y., Sun J., Huang J., Lu Z.X., Regina M.P., Chen J.S., Chen C.M. (2015). Effect of NaFeEDTA-Fortified Soy Sauce on Anemia Prevalence in China: A Systematic Review and Meta-Analysis of Randomized Controlled Trials. Biomed. Environ. Sci..

[208] Forsman C., Milani P., Schondebare J.A., Matthias D., Guyondet C. (2014). Rice Fortification: A Comparative Analysis in Mandated Settings. Ann. N. Y. Acad. Sci..

[209] Hurrell R.F. (2022). Ensuring the Efficacious Iron Fortification of Foods: A Tale of Two Barriers. Nutrients.

[210] Iwuozor K.O., Mbamalu P.S., Olaniyi B.O., Anyanwu V.U., Emenike E.C., Adeniyi A.G. (2022). Fortification of Sugar: A Call for Action. Sugar Tech.

[211] Suchdev P.S., Williams A.M., Mei Z., Flores-Ayala R., Pasricha S.R., Rogers L.M., Namaste S.M.L. (2017). Assessment of Iron Status in Settings of Inflammation: Challenges and Potential Approaches. Am. J. Clin. Nutr..

[212] Fishbane S., Block G.A., Loram L., Neylan J., Pergola P.E., Uhlig K., Chertow G.M. (2017). Effects of Ferric Citrate in Patients with Nondialysis-Dependent CKD and Iron Deficiency Anemia. J. Am. Soc. Nephrol..

[213] Schmidt C., Allen S., Kopyt N., Pergola P. (2021). Iron Replacement Therapy with Oral Ferric Maltol: Review of the Evidence and Expert Opinion. J. Clin. Med..

[214] Gasche C., Ahmad T., Tulassay Z., Baumgart D.C., Bokemeyer B., Büning C., Howaldt S., Stallmach A. (2015). Ferric Maltol Is Effective in Correcting Iron Deficiency Anemia in Patients with Inflammatory Bowel Disease: Results from a Phase-3 Clinical Trial Program. Inflamm. Bowel Dis..

[215] Gómez-Ramírez S., Brilli E., Tarantino G., Muñoz M. (2018). Sucrosomial^®^ Iron: A New Generation Iron for Improving Oral Supplementation. Pharmaceuticals.

[216] Abbati G., Incerti F., Boarini C., Pileri F., Bocchi D., Ventura P., Buzzetti E., Pietrangelo A. (2019). Safety and Efficacy of Sucrosomial Iron in Inflammatory Bowel Disease Patients with Iron Deficiency Anemia. Intern. Emerg. Med..

[217] Giordano G., Napolitano M., Di Battista V., Lucchesi A. (2021). Oral High-Dose Sucrosomial Iron vs Intravenous Iron in Sideropenic Anemia Patients Intolerant/Refractory to Iron Sulfate: A Multicentric Randomized Study. Ann. Hematol..

[218] Cesarano D., Borrelli S., Campilongo G., D’Ambra A., Papadia F., Garofalo C., De Marco A., Marzano F., Ruotolo C., Gesualdo L. (2024). Efficacy and Safety of Oral Supplementation with Liposomal Iron in Non-Dialysis Chronic Kidney Disease Patients with Iron Deficiency. Nutrients.

[219] Maladkar M., Sankar S., Yadav A., Maladkar M., Sankar S., Yadav A. (2020). A Novel Approach for Iron Deficiency Anaemia with Liposomal Iron: Concept to Clinic. J. Biosci. Med..

[220] de Alvarenga Antunes C.V., de Alvarenga Nascimento C.R., Campanha da Rocha Ribeiro T., de Alvarenga Antunes P., de Andrade Chebli L., Martins Gonçalves Fava L., Malaguti C., Maria Fonseca Chebli J. (2020). Treatment of Iron Deficiency Anemia with Liposomal Iron in Inflammatory Bowel Disease: Efficacy and Impact on Quality of Life. Int. J. Clin. Pharm..

[221] Biniwale P., Pal B., Sundari T., Mandrupkar G., Datar N., Khurana A.S., Qamra A., Motlekar S., Jain R., Biniwale P. (2018). Liposomal Iron for Iron Deficiency Anemia in Women of Reproductive Age: Review of Current Evidence. Open J. Obs. Gynecol..

[222] Mohammed N.I., Wason J., Mendy T., Nass S.A., Ofordile O., Camara F., Baldeh B., Sanyang C., Jallow A.T., Hossain I. (2023). A Novel Nano-Iron Supplement versus Standard Treatment for Iron Deficiency Anaemia in Children 6–35 Months (IHAT-GUT Trial): A Double-Blind, Randomised, Placebo-Controlled Non-Inferiority Phase II Trial in The Gambia. EClinicalMedicine.

[223] Tsilika M., Mitrou J., Antonakos N., Tseti I.K., Damoraki G., Leventogiannis K., Giamarellos-Bourboulis E.J. (2023). An Active New Formulation of Iron Carried by Aspartyl Casein for Iron-Deficiency Anemia: Results of the ACCESS Trial. Ann. Hematol..

[224] Meeting Highlights from the Committee for Medicinal Products for Human Use (CHMP) 24–27 March 2025|European Medicines Agency (EMA). https://www.ema.europa.eu/en/news/meeting-highlights-committee-medicinal-products-human-use-chmp-24-27-march-2025.

[225] Scott L.J. (2018). Ferric Carboxymaltose: A Review in Iron Deficiency. Drugs.

[226] Keating G.M. (2015). Ferric Carboxymaltose: A Review of Its Use in Iron Deficiency. Drugs.

[227] INJECTAFER® Approved in the U.S. for the Treatment of Iron Deficiency in Adult Patients with Heart Failure|HFSA. https://hfsa.org/injectaferr-approved-us-treatment-iron-deficiency-adult-patients-heart-failure.

[228] Zoller H., Wolf M., Blumenstein I., Primas C., Lindgren S., Thomsen L.L., Reinisch W., Iqbal T. (2023). Hypophosphataemia Following Ferric Derisomaltose and Ferric Carboxymaltose in Patients with Iron Deficiency Anaemia Due to Inflammatory Bowel Disease (PHOSPHARE-IBD): A Randomised Clinical Trial. Gut.

[229] Cleland J.G.F., Kalra P.A., Pellicori P., Graham F.J., Foley P.W.X., Squire I.B., Cowburn P.J., Seed A., Clark A.L., Szwejkowski B. (2024). Intravenous Iron for Heart Failure, Iron Deficiency Definitions, and Clinical Response: The IRONMAN Trial. Eur. Heart J..

[230] Adkinson N.F., Strauss W.E., Macdougall I.C., Bernard K.E., Auerbach M., Kaper R.F., Chertow G.M., Krop J.S. (2018). Comparative Safety of Intravenous Ferumoxytol versus Ferric Carboxymaltose in Iron Deficiency Anemia: A Randomized Trial. Am. J. Hematol..

[231] Van Doren L., Auerbach M. (2023). IV Iron Formulations and Use in Adults. Hematology.

[232] Intravenous Iron Offers Some Benefits for Some Patients with Heart Failure–American College of Cardiology. https://www.acc.org/About-ACC/Press-Releases/2025/03/30/12/35/Intravenous-Iron-Offers-Some-Benefits-for-Some-Patients-with-Heart-Failure?.

[233] Derman R.J., Bellad M.B., Somannavar M.S., Bhandari S., Mehta S., Mehta S., Sharma D.K., Kumar Y., Charantimath U., Patil A.P. Single-Dose Intravenous Iron vs Oral Iron for Treatment of Maternal Iron Deficiency Anemia: A Randomized Clinical Trial. Am. J. Obs. Gynecol..

